# Mining gene expression data by interpreting principal components

**DOI:** 10.1186/1471-2105-7-194

**Published:** 2006-04-07

**Authors:** Joseph C Roden, Brandon W King, Diane Trout, Ali Mortazavi, Barbara J Wold, Christopher E Hart

**Affiliations:** 1 Jet Propulsion Laboratory, California Institute of Technology, Pasadena, USA; 2 Division of Biology, California Institute of Technology, Pasadena, USA

## Abstract

**Background:**

There are many methods for analyzing microarray data that group together genes having similar patterns of expression over all conditions tested. However, in many instances the biologically important goal is to identify relatively small sets of genes that share coherent expression across only some conditions, rather than all or most conditions as required in traditional clustering; e.g. genes that are highly up-regulated and/or down-regulated similarly across only a subset of conditions. Equally important is the need to learn which conditions are the decisive ones in forming such gene sets of interest, and how they relate to diverse conditional covariates, such as disease diagnosis or prognosis.

**Results:**

We present a method for automatically identifying such candidate sets of biologically relevant genes using a combination of principal components analysis and information theoretic metrics. To enable easy use of our methods, we have developed a data analysis package that facilitates visualization and subsequent data mining of the independent sources of significant variation present in gene microarray expression datasets (or in any other similarly structured high-dimensional dataset). We applied these tools to two public datasets, and highlight sets of genes most affected by specific subsets of conditions (e.g. tissues, treatments, samples, etc.). Statistically significant associations for highlighted gene sets were shown via global analysis for Gene Ontology term enrichment. Together with covariate associations, the tool provides a basis for building testable hypotheses about the biological or experimental causes of observed variation.

**Conclusion:**

We provide an unsupervised data mining technique for diverse microarray expression datasets that is distinct from major methods now in routine use. In test uses, this method, based on publicly available gene annotations, appears to identify numerous sets of biologically relevant genes. It has proven especially valuable in instances where there are many diverse conditions (10's to hundreds of different tissues or cell types), a situation in which many clustering and ordering algorithms become problematic. This approach also shows promise in other topic domains such as multi-spectral imaging datasets.

## Background

Bioinformatics has placed much emphasis on using various unsupervised clustering techniques as a means to understand the information present in gene microarray expression datasets. Clustering techniques produce a rich taxonomy of results by defining groups of genes that act more or less similarly across a number of experimental conditions. The diverse approaches to clustering genes by expression levels include k-means [1], self-organizing maps [2], hierarchical algorithms [3,4] and probabilistic models [5]. Some approaches permit clustering of the conditions as well [6-8]. Based on co-expression, genes that comprise individual expression clusters are often postulated to be co-regulated, and to the extent that this hypothesis is correct in any specific biological situation, the gene cluster definitions can offer key insights into gene regulatory network (GRN) structure and function.

Another common data mining task is to try to identify small sets of genes that can serve as effective predictors of disease diagnosis or prognosis. While clustering at its best is good at finding sets of genes that are similarly expressed across all conditions within a dataset, many issues (e.g. selection of K, stochastic effects, and "noise" from large numbers of genes that change little over most of the conditions) can prevent clustering from successfully highlighting small groups of interestingly co-expressed genes [9,10]. This often encountered problem is addressed in part by hierarchical phylogenetic ordering algorithms such as average linkage in Mike Eisen's cluster program [3], but the information biologists seek regarding shared sub-patterns of co-expression can be obscured by both algorithmic and visualization constraints. The algorithmic limitations in hierarchical clustering confound and "cover" the presence and organization of smaller and more specific gene groups that are similar across only a subset of conditions within the larger dataset. In any case, biologists generally subjectively define a cluster of genes from such phylogenetic trees based largely on human pattern recognition. Finally, nothing inherent in the clustering approach helps to direct a biologist to which cluster is interesting or relevant. Instead, biologists generally take the path of focussing on a group of genes exhibiting a pattern of expression that supports a specific hypothesis, or search for a known gene or genes of interest within a cluster to form an explanation for others in the cluster.

Support vector machines have been shown to be useful for identifying small sets of related and predictive genes [11-14], but represent a supervised learning approach which requires one to first define a set of known positive examples, a set of known negative examples, and a specific covariate to predict. We wanted an unsupervised algorithm that would help us to find relationships and structure in the data that is more specific than what clustering algorithms usually deliver, yet is hypothesis independent. We found it efficient and useful to use as an independent starting tool a very direct approach based on principal components analysis (PCA, see Methods section). A virtue is that this approach is computationally efficient for very large datasets, especially compared with most clustering algorithms, but is also applicable to much smaller ones. It allows one to directly explore each of the independent and diverse sources of variation present within a gene expression dataset and to subsequently identify the specific genes that vary the most, together with the conditions in which they vary.

Unlike conventional clustering and ordering algorithms, this PCA based approach permits a gene to be highlighted and grouped as influential in multiple condition sets, whereas in cluster membership a gene is typically assigned to one unique cluster. The "single cluster assignment" quality of traditional clustering and ordering algorithms is problematical because it tends to hide commonality of expression that is restricted to a small, interesting, and often entirely unpredicted subgroups of tissues, cell types, treatments or other condition types. This situation, perhaps because of inherent properties of gene network structure, will arise increasingly as the number and diversity of conditions represented in expression datasets increases.

The clustering method of Barkai et al. [15,16] addresses this issue of multiple membership in a different way, by using randomly-selected gene sets to iteratively search for and refine self-consistent groups. Their approach, which is related to PCA through singular value decomposition (SVD), also permits genes to be assigned to multiple "expression modules." In contrast to the method presented here, there is no provision for correlating modules with covariate data.

The use of principal components analysis presented here differs from other recent applications in gene expression analysis. PCA is most commonly used in as a means of dimensionality reduction prior to clustering [7,17] or prior to classification [18,19]. It is also used to visualize or confirm clustering results [19-21]. In contrast, our use of PCA aims to find, then examine, and where possible, generate hypotheses to explain individual principal components. In this manner, we build on the observations by Hilsenbeck [22] Raychaudhuri et. al. [23] who used PCA to gain insight into the underlying factors present in the Chu et. al. yeast sporulation experiments [24]. Wall et. al. [25] introduced a novel use of singular value decomposition (SVD) for gene expression analysis that identifies non-exclusive gene groups, and Selaru et. al. [26] illustrated the potential of PCA to detect molecular phenotypic bases that correspond to relevant clinical or biological features of human tumors. Their approach identifies a subset of principal components that correlate well with known covariates. Here we introduce methods that extend beyond producing gene groups and observing a few principal components. Our methods provide a path for systematically analyzing each principal component by identifying the genes most influential in defining a particular principal component and the conditions in which those influential genes vary significantly. Finally, we describe methods which aim to explain each principal component's observed variance in terms of the condition variables deemed most likely to be driving the variance. We also introduce a software package that implements these methods. These methods and our software package provide automated and objective way of doing what a biologist naturally tries to do through inspection and pattern recognition.

## Implementation

We have developed a Python package to implement the PCA interpretation capability described in detail in the Methodology section. This PCA analysis package has been added to CompClust developed previously [9,27]. The combined packages allow one to cluster, classify and visualize numeric datasets that have discrete or numeric annotations (referred to as labelings, or labelled datasets), and to compare labelings with confusion matrices and metrics such as normalized mutual information (NMI) [28]. This PCA analysis tool (including the complete results for the dataset analysis described in the Results section) has also been made accessible through the CompClustWeb web-based interface [29]. Our software makes use of data manipulation and graphical plotting using the matplotlib package [30], and the statistics are generated using the rPy package [31] and Gary Strangman's Python stats package [32].

The web-based front-end permits users to get a complete report on the interpretation of each principal component, including interactive PCA projection plots with the principal component's extreme genes (PCEGs) highlighted; ranked lists of the PCEGs with detailed annotations; interactive significance-ordered gene set trajectory plots that permit users to drill down to the individual gene level; similarly ordered condition reports ordered by expression difference and grouped by significance, including covariate info (with significantly correlated covariates highlighted); and finally a report of any suggestive covariates that are well correlated with the significant column grouping, including the confusion matrices and/or plots of statistics scores to back up the conclusions. All principal component analysis and results generation is implemented in a Python package so that analyses of large datasets can be executed in a batch mode rather than through the graphical interface. Further, the software that implements the CompClustWeb interface is provided within the CompClust package, so a software developer can create his or her own CompClustWeb server to review results of their PCA interpretation.

## Results

### Application to microarray expression data, Case 1: GNF human data

We obtained gene expression data from the Genomics Institute of the Novartis Research Foundation ("GNF") Gene Expression Database via their SymAtlas web site [33,34]. The dataset is a challenge for most clustering algorithms because it contains 158 tissue samples hybridized to two Affymetrix microarray chips: U133A and GNF1H. The dataset combines the measurements of these chips to provide a total of 33,689 unique probe identities across the 158 tissue samples. Expression data are signal intensities estimated by Affymetrix Microarray Suite v5. For our analysis we used the log base 2 of the expression signal, and included data for all tissues and probes (noting that absent and present calls were not provided with the signal intensities). We applied our principal components analysis tool to generate interpretations for each of this dataset's 158 principal components.

**Figure 1:**
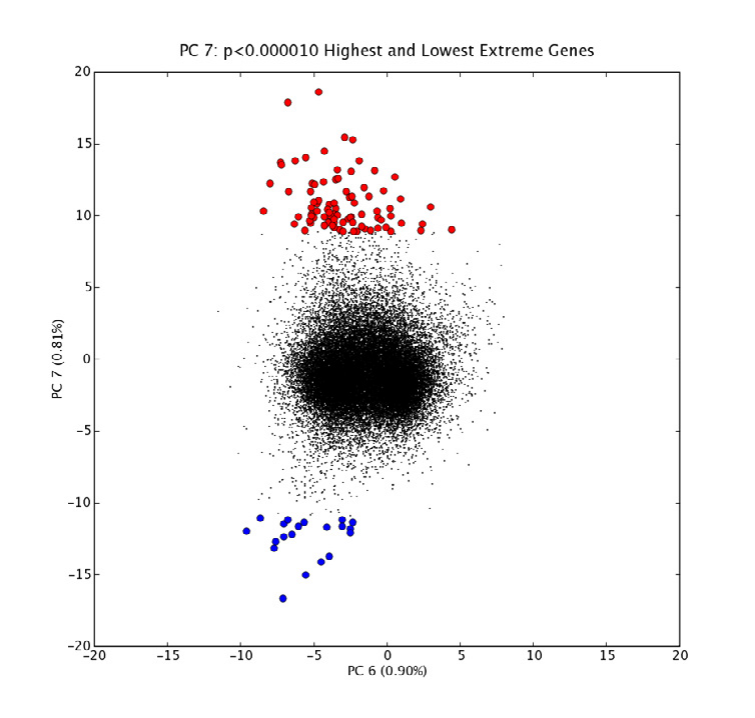
**GNF PC7 High and Low Extreme Gene Probes**. Scatter plot of N = 33689 probe expression levels projected onto PC_6 _vs. PC_7 _space with high and low extreme gene sets *H_7 _*(red points, *n *= 88) and *L_7 _*(blue points, *n *= 20); extreme genes selected at *extremeThresh *= 0.00001. The extreme genes in *H_7 _*are listed in Table 2.

As detailed in the Methodology section, for each principal component we identified a set of gene probes occupying the high and low extremes of that principal component's axis (we refer to these as principal component extreme genes, or PCEGs). One can adjust parameters to recover smaller and larger numbers of PCEGs per component by specifying either a likelihood threshold or an explicit number of PCEGs. The PCEGs are those probes having the most highly weighted values for that principal component, selected because they stand out from the others, they influence the principal component's direction, and thus they warrant further investigation. We selected probes with likelihoods less than *extremeThresh *= 0.00001, which yielded on average 20 low and 20 high extreme genes per principal component, though the sets sizes do vary considerably (μ = 18.9, σ = 17.2). Next we identified the tissues in which the high PCEGs showed significantly different expression than the low PCEGs. Visualizations produced include scatter plots of the extreme genes in PCA sub-spaces (PC *N*-1vs. PC *N*), and extreme gene trajectories in original tissue order as well as with tissues ordered by decreasing difference of mean of high PCEGs and mean of low PCEGs. The latter trajectory plot emphasizes how the extreme genes for a principal component show a pattern of expression that imposes a partitioning of tissues. It is left to human interpretation to examine the extreme genes and the tissue partitioning exposed by each principal component, and thus to build hypotheses that attach meaning to the sources of variation. The percentage variance explained by the top 50 principal components is provided in Table 1. Example results for two illustrative principal components, PC7 and PC21, are shown in Figures 1 through 4 and in Tables 2 through 5, and are discussed below. The complete analysis results generated for PC7 are provided as an example in the supplemental files: [see Additional file 1], [see Additional file 2], [see Additional file 3], [see Additional file 4], [see Additional file 5], [see Additional file 6]. Our supplemental materials web site [35] contains the complete collection of PCA interpretation results generated for these dataset for all principal components, as well as results of a comparable analysis done at *extremeThresh *= 0.001 which yielded larger PCEG sets.

We addressed the question of biological and statistical significance of PCs and the sets of extreme genes identified. Each set of high and low extreme genes from each principal component was tested for Gene Ontology (GO) statistical enrichment when compared to the human GO annotations from NCBI's loc2go dataset using the hypergeometric to calculate the p-value of each GO term. Terms that were enriched for a particular PC at 1% significance threshold and that were still significant following a Bonferroni correction for multiple hypothesis testing as described in [36] are reported as enriched (see Table 6). 26 of the top 42 PCEG lists, derived using a stringent cut-off of 0.00001, produced significant GO enrichments; no PCEG sets beyond PC42 showed significant enrichment. As discussed below, many of the significant results showed obvious biological coherence and relationships to the specific samples associated with the PC of origin. This argues that PCs containing less than 1% of the total variation in this dataset are still relevant and point to coherent and important gene sets and their related samples.

Relationships between extreme gene sets and the corresponding sets of driving samples could be discerned for many PCs. The Methodology section presents a way to additionally correlate each principal component's sample partitioning with any available sample covariates. Although some human sample covariate information is provided in our test case, the GNF human expression dataset is not amenable to this additional layer of analysis because multiple subject's RNA samples were pooled prior to amplification and array hybridization. However, a second publicly available dataset with rich covariate information is presented below.

### Application to microarray expression data, case 2: human diabetes

We acquired a Human diabetes expression dataset [37] from the Broad Institute Cancer Program dataset repository [38] along with the corresponding phenotype covariate data, and applied the filtering step as they described to produce a set containing 10,983 probes across 43 samples. Tissue samples were skeletal muscle biopsies from 3 diagnosis groups: normal glucose tolerance (NGT, *n *= 17); impaired glucose tolerance (IGT, *n *= 8); and Type 2 diabetes mellitus (DM2, *n *= 18). We used our PCA interpretation software to perform an unsupervised analysis of the DM2 vs. NGT subset (as that subset is comparable to the previous published result). As described in the Methodology section, PCEG sets were determined using an *extremeThresh *likelihood threshold of 0.001, which yielded about 50 high and 50 low extreme genes per principal component. For each principal component *N*, samples were partitioned in to *UP*_*N*_, *FLAT*_*N *_and *DOWN*_*N *_sample sets on the basis of PC-*N *extreme high and extreme low expression differences. The supplemental materials contain PCA interpretation results for all 35 principal components, as well as results of a comparable analysis done at *extremeThresh *= 0.0001 which yielded smaller PCEG sets (see [35]).

**Figure 5:**
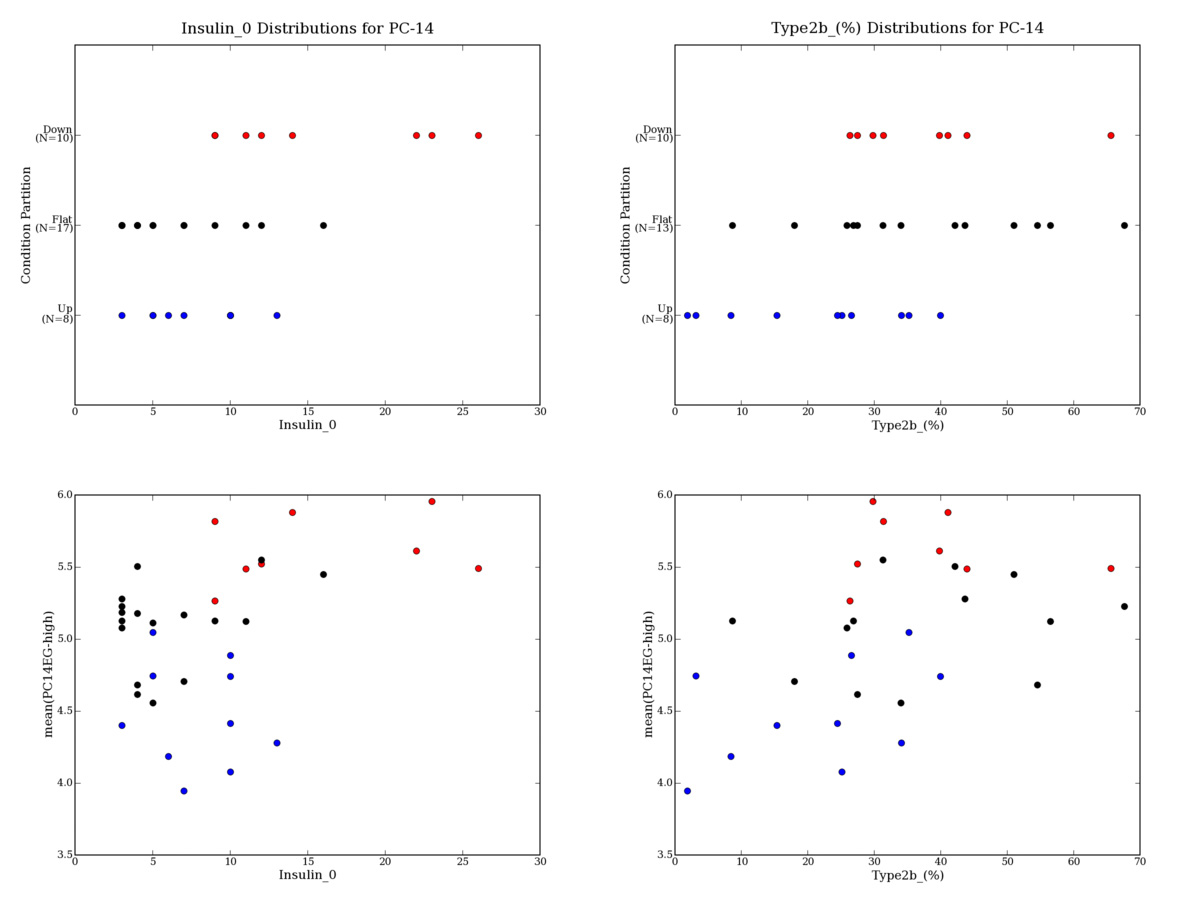
**Diabetes PC14 Sample Partitioning is Correlated with Certain Covariates**. When sufficient covariate data is available (a number of the measurements are missing for certain covariates), covariate distributions are compared across partitions and significant differences are reported (as in Table 7). When a covariate is identified as significantly correlated with a principal component's sample partitioning, covariate distribution plots can be generated to further investigate and evaluate the apparent relationship. For example, the diabetes dataset's PC14 extreme genes partition the samples into *UP_14 _*(*n *= 8), *FLAT_14 _*(*n *= 17) and *DOWN_14 _*(*n *= 10) based on their expression patterns. PC14's *UP_14 _*vs. {*FLAT_14_*+*DOWN_14_*} sample partitioning appears related to the Insulin_0 measure (*sig *= 0.001), and the {*UP_14_*+*FLAT_14_*} vs. *DOWN_14 _*partitioning appears related to Type2b_(%) (*sig *= 0.008). The mean expression of the PC14EG-high genes appears modestly correlated with Insulin_0 (*r *= 0.411) and with Type2b_(%) (*r *= 0.467). *UP_14 _*samples are in red, *FLAT_14 _*are black, and *DOWN_14 _*are blue.

This dataset contains more than 50 covariates, which provides the opportunity to interpret each principal component by searching for covariates that correlate well with expression patterns in the PCEG sets. As described in the Methodology section, we asked if any of the covariate annotations are well correlated with the partitioning of samples into *UP*_*N*_, *FLAT*_*N *_and *DOWN*_*N *_sets. Covariate distributions were compared across different partitions (when sufficient data was available) and any significant trends identified were recorded (see Table 7). For covariates identified as significantly correlated with a principal component's sample partitioning, covariate distribution plots were generated to further investigate and evaluate the apparent relationship. For example, Figure 5 illustrates that PC14's *UP_14_*, *FLAT_14 _*and *DOWN_14 _*sample partitions appear to be significantly related to two covariate measurements: Insulin_0 (*sig *= 0.0010); and Type2b_(%) (*sig*= 0.0077). The Pearson's correlation between the mean expression for the PC14EG-high set and Insulin_0 and Type2b_(%) covariates are *r *= 0.411 and *r *= 0.467 respectively.

## Discussion

This PCA-based data-mining tool highlights specific patterns of expression and associates them in a convenient way with the genes and samples responsible for those patterns. Some associations in the first few principal components (PCs) of the GNF set reflect major features in the data that are expected. This includes the global high and low constitutive expression profiles of PC1 (67% of variance in the GNF dataset). A component similar to this is often the first or second PC in Affymetrix array datasets. GNF PC3, in contrast, highlighted brain/neuronal tissues, which we expected in this dataset because there are many more samples from brain regions than from any other tissue, and there are thousands of genes that are expressed in a general brain pattern. The GO enrichment analysis associated with PC3lowEGs confirmed this impression by identifying neurogenesis, central nervous system, and synaptic terms as significantly enriched for PC3EG (Table 6).

We asked if principal components that account individually for small fractions of variation in the data are likely to be significant. Conventional practice generally ignores principal components accounting for a few percent, or less, of total variation, on the assumption that such minor components are most likely dominated by noise. We believe this assumption, that all of the PC's accounting for small fractions of data should be ignored because they are artefact, to be wrong in the context of our analysis. We believe this, in part, because in our analysis we find many PCEGs for minor components are statistically enriched (Table 6). Further, computational experiments using randomized data fail to produce any significant enrichments. This GO enrichment analysis, it should be noted, tends to underestimate the fraction of gene sets that are significant. This is a known artefact in instances where the input gene number (here those passing the selected rank sum threshold) is small, and its effects are exacerbated by the fact that GO annotations for human are still very much in a building phase. Many genes that will eventually be associated with GO terms are not yet entered. This means that reducing the threshold modestly, and therefore increasing the gene number, can uncover additional significant GO term enrichment in some of these PCs. For complete results of GNF analysis at *p *< = 0.001 see our supplemental materials web site [35].

Viewed from a biological perspective, this PCA mining revealed several different classes of relationships. GNF PC21 is a good example of a component that highlights a coherent gene set and its corresponding tissues, many of which would also be grouped together by conventional clustering algorithms. This is true even though PC21 accounts for just 0.25% of total variation. The PC21EG-low set (defined at *p *< = 0.00001) was enriched in a statistically significant way for five different GO terms (Table 6), and these terms (myogenesis, muscle contraction, etc.) tell a simple and internally consistent story about muscle development and function. The top tissue samples driving PC21-low are skeletal muscle, tongue (also composed largely of striated muscle), heart and thyroid (which includes a population of myoid cells). Most top-ranked genes in this PCEG set are so specific for striated muscle that they would also appear together in conventional clusterings, although many clustering approaches become technically problematic with datasets of this size. However the PC21EG-low list differs from a conventional muscle cluster because it also includes some genes that are partly associated with muscle and partly associated with other tissues. PP1R1A, a regulatory subunit of protein phosphatase1, is such a gene. A role for it in striated muscle is suggested based on its coherent presence in tongue, skeletal and cardiac samples, even though it might well not have been seen in this light by standard clustering.

The second example is GNF PC7, which accounts for 0.81% of variation. It illustrates a different kind of biological relationship that more strongly distinguishes results of PCA mining from classical clustering. Top extreme genes associated with PC7 by inspection turn out to be a "who's who" of extracellular matrix components (a specific subset of fibronectins, collagens, laminins plus matrix associated proteins like *MFAP5*, *MGP*, *LUM*; regulatory molecules that mediate stability and function of those matrix components (thrombospondin, *SPARC*, *ADAMTS1*, *Plod2*); and matrix associated signalling and matrix associated signal modulators (insulin like growth factor binding proteins 7, 8 and 10; *Sema3c*). GO analysis confirms what inspection of the top PC7EGs suggested: namely that a set of extracellular matrix components are expressed in these driving tissues. It is instructive to look at the individual expression profiles for these genes directly at the GNF website and also in aggregate, as represented in the tissue (conditions) list in Table 2. The most prominent contributing tissues associated with high expression of these genes are informative specifically because they do not constitute a group that would have been selected *a priori *as a coherent set based on known tissue function or shared developmental origin.

This is useful because a biologist interrogating the GNF database would not likely have constructed a query combining adipocytes, smooth muscle, bronchial epithelium, nor would one expect traditional clustering algorithms to place these genes so close to each other as to catalyze the same observation. Similarly, conventional ordering algorithms would not have placed them adjacent to each other because other parts of their expression profiles, containing different genes than those in PC7, would dominate their positions. And concerning genes, they have, in addition to commonalities of expression highlighted by PC7, differences from each other in additional diverse tissues not highlighted by PC7. The PCA grouping gives impetus and a necessary starting gene list to search for one or more factors or regulatory RNAs with a similar expression pattern, or to search for a shared and perhaps evolutionarily conserved cis-acting DNA sequence motifs. It is unlikely that these working hypotheses would have been arrived at easily by widely used methods of gene expression analysis.

The diabetes dataset offered us an opportunity to add more value to principal component interpretations by searching for covariates that appear correlated at some significance level. The relationships highlighted between covariates and principal components are suggestive, but not conclusive by themselves. Rather, they provide hypotheses that a researcher may wish to further investigate. While we have not delved deeply into this dataset, we believe that a number of the principal components are highlighting a number of meaningful sources of variation present. It is not clear whether this set should exhibit the same proportion of meaningful principal components as the GNF dataset, as by design the NGT vs. GM2 dataset does not contain substantial diversity of samples. Likewise, the selection of covariates was focused on a narrow set of measurements selected to be indicative of diabetes status, and so many covariates are redundant. We anticipate this tool will be maximally useful in cases where datasets are rich in both sample complexity and diversity of covariates.

## Conclusion

Results presented above show that this PCA-mining approach can guide a user to biologically significant observations that both complement and reinforce those from conventional clustering analysis. The software package and web interface make this style of microarray analysis straightforward and accessible.

We have applied this to four additional microarray datasets (as yet unpublished) and to one multi-spectral imaging dataset. In each case we found the interpretations that the tool presented to be useful. In general, it seems that the top few principal components identify very broad characteristics of the data. Digging to the deeper components that comprise smaller but more particular substructure in the data leads to more subtle but often meaningful observations, many being complementary to standard clustering.

With respect to the top few components, PC1 is usually the approximate diagonal through the sample/condition space, explaining the overall variation in absolute expression level. For some other datasets we have noticed that the top few PCs can also highlight effects of preprocessing normalization steps or global data quality issues. This means they do not necessarily expose the most important biological variation. Thus, in one microarray dataset not shown here, PC2 was found to be extremely well correlated with a measure of quality of samples, as reflected by the percent of Affymetrix probes called present. Given this evidence of data quality effects comprising a major source of variation over the entire dataset, one might be motivated to remove the major offending conditions, and then repeat the PCA interpretation on the remaining conditions (columns). The idea is that an independent source of variation might be obscured by more dominant signals or noise present in the data from the offending condition.

Our experience thus far leads us to think that this PCA interpretation method will contribute to microarray expression analysis, as one part of a panel of methods that are sensitive to different features in a dataset, such as sample number, gene number, and distribution of variation across the samples. The PCA method should be especially useful for large, complex datasets that offer rich variation among many samples. What is certain is that there are almost always multiple sources of variation in a dataset and that in any specific study their nature and relative strength is informative, whether the origin is an easily-understood biologic one, a technical one, or a poorly-understood but nonetheless biologically pertinent one.

We are continuing to explore ways to improve our methodology and software package. We anticipate further advances will come with software infrastructure improvements to permit covariate analyses of both column (sample) covariates and row (gene) covariates. The CompClust dataset labeling capability [27] allows a user to attach diverse and numerous labelings to rows or columns. For example we can pull in additional row (gene probe) annotations such as Gene Ontology (GO) functional groups. Beyond explicitly comparing the NMI significance of specific row partitionings for discrete covariates, we plan to add routines to CompClust to automatically indicate when a group of genes are found to be enriched in specific GO categories (as was done in our analyses above), and more generally to handle large, discrete, multi-valued distributions of values. Our use of NMI treats discrete covariates as discrete random variables that can have at most a single value per condition, and so does not optimally address issue of multi-valued discrete random variables (e.g. GNF data has covariate "concomitant medications" with values like "aspirin", "tylenol", and "aspirin & tylenol"). We are considering more elaborate extensions of mutual information or alternatives that might be able to take further advantage of such multi-valued entries.

## Availability and requirements

The PCA interpretation software is implemented as one component of the CompClust Python package [9,27], which is freely available for non-commercial use. The software capability is also accessible through the CompClustWeb web-based interface [29]. The software that implements the web application is also included within the CompClust software distribution.

Project name: the PCA interpretation component of CompClust

Project home page: 

Operating system(s): platform independent (Windows, Linux, Mac OS X)

Programming language: Python

Other requirements: Python 2.3 or higher (and some free Python packages)

License: MLX Public License 1.0 (non-commercial use allowed)

Use by non-academics: licence needed

We recommend that interested researchers use the web-based application, CompClustWeb, from any platform to review the PCA interpretation results for the GNF human gene expression and Broad Institute human diabetes expression data sets. We have written a CompClust PCA interpretation tutorial that demonstrates how to use CompClust's programming interface to generate PCA interpretations. Following the tutorial requires that CompClust and its Python prerequisites be installed. We created an easy-to-use CompClustShell installer for Windows that provides everything needed. For other operating systems (e.g. Linux & OS X) we recommend that a software developer or system administrator help with the CompClust source code installation. We are working to simplify installation and plan to provide user-friendly installers for other operating systems in the near future.

## Methodology

We have developed the following algorithm for identifying and analyzing multiple independent sources of variance present within multi-dimensional sample datasets, in particular those that are produced by gene microarray expression experiments. The overall approach can be summarized as follows: 1) perform principal components analysis of the dataset; for each principal component: 2) identify the most extreme gene probes (those with the highest or lowest weighting) for that principal component; 3) identify and group any conditions in which those extreme probes vary significantly; 4) identify any condition covariates that correlate well with the condition grouping. By extending the interpretation of each principal component from extreme genes (rows) to ordered groups of significant conditions (columns) and further to identifying statistically significant correlations with column covariates, we attempt to make full use of the available data, in an objective and data-driven way, to analyze and provide meaningful interpretations of the diverse sources of variation present within the dataset.

### Determine the principal components of the dataset

Our dataset *D *consists of *nc *columns (e.g. tissue samples or conditions) and *nr *row vectors (e.g. gene probes), each row vector *x_i _*∈ ℜ^*nc *^where *i *∈ [1, *nr*]. Such a dataset is usually represented as a two-dimensional *nr *× *nc *matrix (where *nr *> *nc*). The dataset may optionally have *nk *supplemental covariate annotations *C *associated with each row or column. Each annotation *C*_*k *_where *k *∈ [1, *nk*] can be either discrete (e.g. sex) or continuous (e.g. age), to permit the association of one discrete value per column (e.g. values male or female), or one continuous value per column (e.g. values 12, 16, or 42).

Our procedure starts by employing principal components analysis (PCA) to sequentially identify a series of new basis vectors or axes *PC*_1_, *PC*_2_, ...*PC*_*nc *_in the high-dimensional column space ℜ^*n *^that are each aligned sequentially to capture the most as-yet unexplained variance. This is accomplished by applying the numeric procedure singular value decomposition (SVD) to the covariance matrix of *D*, *cov(D)*, to produce the decomposition *cov*(*D*) = *USV^T ^*that contains the eigenvectors of *cov*(*D*) in the columns of *U *and eigenvalues in the diagonal of *S *such that the eigenvalues are sorted by descending size. Each covariance eigenvector, or principal component *PC*_1_, *PC*_2_, ...*PC*_*nc*_, explains a fraction of the total variance contained in the dataset, and each principal component *PC*_*n+1 *_is orthogonal to the previous principal component *PC*_*n*_. such that they define the basis of a new vector space *P*. These results are made available to the users in the form of *nc *plots, one for each of the principal component vectors, as well as a plot of the singular values contained in the diagonal of *S *to indicate the relative amount of variance each component explains.

### Identify extreme gene probes for each principal component

Next, we project each data point *x_i _*(corresponding to a gene probe, or row vector) into the new coordinate system by *P *= *DU*, effectively rotating the entire data point set *D *into the new principal component axes space, producing the rotated data set *P*. Each data point *p_i _*in the rows of *P *corresponding to *x_i _*has a coordinate for each principal component axis that describes where the data point *p_i _*lies when projected along each axis *PC*_1_, *PC*_1_, ...*PC*_*nc*_. For each principal component *PC*_*n *_(*n *∈ [1, *nc*]) we select a set of data points from each end of that principal component axis- these are the extreme points for *PC*_*n*_, called the principal component extreme genes, or PCEGs for convenience. The PCEGs can be identified and ranked in one of two ways: by identifying points having a low probability (*p *< = *extremeThresh*) of belonging to a Gaussian fit to the distribution of points along the *PC*_*n *_axis, or by taking a fixed number of *nExtreme *points at each tail of the distribution. *H*_*n *_is the resulting set of data points having the highest coordinate values for *PC*_*n*_, and *L*_*n *_is the resulting set of data points having the lowest coordinate values for *PC*_*n*_. These high and low extreme gene point sets are informative in and of themselves because they represent the most extreme of the data points along a principal axis of variation. As such, the high and low PCEG sets are some of the primary outputs generated by our procedure. We use the term "extreme" in a very general sense, in that the points stand out because they are far from the main distribution. We do not mean to imply that such points are either biologically relevant or nuisance data that should be removed; rather we are interested in these points in an unbiased way. By further analyzing their pattern of expression in the original axes we hope to gain a better understanding of their possible biologic significance.

### Identify significant conditions for each principal component

The extreme gene points comprising *H*_*n *_are located near one edge of the high-dimensional cloud of points, and points *L*_*n *_are near the opposite edge. Thus, points *H*_*n *_are likely to have coordinate values that are maximally different from points *L*_*n *_in a subset of the original column space coordinate system. Our procedure next seeks to identify in which of the original columns (the original axes or dimensions) we find the greatest difference of values for points *H*_*n *_versus points *L*_*n*_. We do this by comparing distributions of values in *H*_*nj *_versus *L*_*nj *_for each of the original columns *j *(*j *∈ [1, *nc*]). A two-sided Wilcoxon rank sum test is used to estimate the likelihood that these two sets of values are drawn from the same distribution [39]; the resulting p-values for each column are used to rank order and group the columns, rather than as actual probabilities. Columns having a likelihood less than a user-defined significance level *test1Thresh *are identified and placed into one of two column sets: *UP*_*n *_for those where column *j *has *mean*(*H*_*nj*_) > *mean*(*L*_*nj*_), and *DOWN*_*n *_for those columns *j *where *mean*(*H*_*nj*_) <*mean*(*L*_*nj*_). Remaining columns that do not show significant variation are placed in the column set *FLAT*_*n*_. The column sets are also meaningful outputs of our procedure, as *UP*_*n *_and *DOWN*_*n *_describe the groups of columns in which the extreme genes *H*_*n *_and *L*_*n *_vary significantly. Our procedure can output these columns and column sets in various orders simply as an aid to human interpretation, including: original column order; grouped by set and within set ordered by the Wilcoxon p-value significance; ordered by mean difference, *mean*(*H*_*nj*_) – *mean*(*L*_*nj*_); or ordered by the eigenvector column loading. Taken together, the PCEG point sets and significant column sets should provide valuable insight to researchers wishing to interpret each of the sources of variation identified by the principal components procedure.

### Interpret each principal component using covariate annotations

When provided additional covariate annotations *C*, the procedure seeks to determine which, if any of the annotations *C*_*k *_are well correlated with the partitioning of columns into the sets {*UP*_*n*_, *FLAT*_*n*_, *DOWN*_*n*_}. A discrete annotation *C*_*k *_containing *m *unique values *V_1_*, *V_2_*, ... *V*_*m *_also defines a partitioning of the columns {*KV_1_*, *KV_2_*, ..., *KV*_*m*_} where *KV_1 _*is the set of columns that share the value *V_1_*, *KV_2 _*are those that share value *V_2_*, and so on. An information theoretic measure known as normalized mutual information (NMI) [28] describes the degree to which two discrete random variables share information. When there is high mutual information, knowing the value of one of the variables should be useful predictor of the other variable. (See [9] for a description of the merits of NMI in terms of clustering and understanding the relationships between clusterings.) We construct the *3 x m *confusion matrix to compare the {*UP*_*n*_, *FLAT*_*n*_, *DOWN*_*n*_} column partitioning with the {*KV_1_*, *KV_2_*, ..., *KV*_*m*_} partitioning and calculate an NMI score between the partitionings. Because the usual NMI score is not symmetric (i.e. NMI(*r*,*c*) ≠ NMI(*c*,*r*)), we use a variant that we refer to as the average NMI score, which is simply the average of the NMI of the confusion matrix and the NMI of the transpose of the confusion matrix. Those covariates *C*_*k *_having an average NMI greater than a user defined threshold *nmiThresh *are added to the set of significant covariate annotations *A*_*n*_.

We apply a different approach when evaluating the *C*_*k *_that are continuous covariates. We need to assess whether each principal component's {*UP*_*n*_, *FLAT*_*n*_, *DOWN*_*n*_} column partitioning correlates with each *C*_*k *_distribution of values. We can separately score three different partitioning schemes, *UP*_*n *_vs. *DOWN*_*n*_, {*UP*_*n*_+*FLAT*_*n*_} vs. *DOWN*_*n*_, and *UP*_*n *_vs.{*FLAT*_*n*_+*DOWN*_*n*_}, by determining if *C*_*k*_'s value distributions differ significantly across the partition. E.g. does *C*_*k *_within *UP*_*n *_have a different distribution than *C*_*k *_within *DOWN*_*n*_? Next, does *C*_*k *_within {*UP*_*n *_+ *FLAT*_*n*_} have a different distribution than *C*_*k *_within *DOWN*_*n*_? Finally, does *C*_*k *_within *UP*_*n *_have a different distribution than *C*_*k *_within {*FLAT*_*n*_+*DOWN*_*n*_}? For each partition scheme we again use a two-tailed Wilcoxon rank sum test, including the small sample adjustments when sample size is less than 10, to determine whether the covariate's value distributions on each side of the partitioning differ significantly from each other. A *minSetSize *parameter can be specified as desired to reduce false positives when set sizes are very small, e.g. when comparing a distribution of 2 values vs. 7 values. Thus we calculate the Wilcoxon p-value for three partitionings of columns: *UP*_*n *_vs. *DOWN*_*n*_, {*UP*_*n*_+*FLAT*_*n*_} vs. *DOWN*_*n*_, and *UP*_*n *_vs. {*FLAT*_*n*_+*DOWN*_*n*_}. Those covariates having a p-value less than the user defined threshold *test2Thresh *for any of the three partitionings are added to the set of significant covariate annotations *A*_*n*_.

Upon completion of the covariate analysis, covariates in the set *A*_*n*_, that previously met user-controlled significance thresholds are reported by the software. Covariate reports provide the following information: For discrete-valued covariates, corresponding confusion matrices and average NMI scores are reported; for continuous-valued covariates, the three *Wilcoxon *p-values are reported together with supporting plots illustrating the covariate distributions. There is no guarantee that any covariates will be significantly related to a principal component. Conversely, spurious relationships might be reported, especially in the case of small numbers of samples due to small column partitions. The tool simply points to those covariates related to a principal component that also satisfy a user-controlled significance threshold. It is up to the investigator to consider these hypotheses and to confirm the interesting ones through further investigation.

### Terminating condition

We have shown that some large-scale expression datasets have biologically pertinent structure that is revealed by deep PC analysis that goes well beyond the first few principal components. However there are limits to the depth of mining and these limits depend on both size and character of the dataset. In all cases, the last principal component is not free to seek a source of variation because it must be orthogonal to all prior *nc-1 *components. To some degree that is also true of some portion of latter principal components that explain ever-diminishing fractions of the variance. We suggest that a natural terminating condition exists: When a principal component cannot find any columns in which the extreme gene sets show significant differences, there is no need to proceed to subsequent principal components. We observe that this condition is often not met because the extreme genes are typically differentially expressed in at least a few of the original columns (the original axes or dimensions), even for the most minor principal components. We also observe that variants of a dataset (e.g. representative column subsets) can affect the relative ordering, but not the existence, of multiple factors or sources of variation that are reflected in the minor principal component regime. We may therefore choose to investigate all principal components, but do so with the expectation that minor principal components will describe increasingly subtle sources of variation, which can, and often do, include noisy processes inherent in the data source.

## Authors' contributions

JR and CH conceived the methodology of exhaustively analyzing and interpreting principal components, in particular how to identify extreme genes and significant conditions, and how to automate correlating these conditions with covariates to aid interpretation. JR, BK, DT and CH carried out the software development. JR carried out the initial PCA interpretation studies and drafted the manuscript. BK and DT performed additional PCA analyses and results interpretation. AM performed the gene set GO term enrichment analysis. BW conceived of the GNF dataset interpretation study, participated in its design and results interpretation, and helped to draft the manuscript. All authors read and approved the final manuscript.

## Supplementary Material

Additional File 1 The supplemental files provided with this publication are only a representative set of those generated by the PCA interpretation software. The complete collections of PCA interpretation results for both the GNF and diabetes datasets are provided as a supplement to this publication at [35].
Tab-delimited text file listing the high and low extreme genes for PC_7_, including PC_7 _coefficient and additional GNF gene annotations: ProbeId, Name, Aliases, Description, Function and Protein Families. Click here for file

Additional File 2 The supplemental files provided with this publication are only a representative set of those generated by the PCA interpretation software. The complete collections of PCA interpretation results for both the GNF and diabetes datasets are provided as a supplement to this publication at [35].
Tab-delimited text file listing the conditions that are up, flat and down for PC_7_, ordered by decreasing difference of means. Click here for file

Additional File 3 The supplemental files provided with this publication are only a representative set of those generated by the PCA interpretation software. The complete collections of PCA interpretation results for both the GNF and diabetes datasets are provided as a supplement to this publication at [35].
Trajectory plot of the PC_7 _eigenvector, or "eigen-condition". Click here for file

Additional File 4 The supplemental files provided with this publication are only a representative set of those generated by the PCA interpretation software. The complete collections of PCA interpretation results for both the GNF and diabetes datasets are provided as a supplement to this publication at [35].
Scatter plot of gene probe expression levels projected onto PC_6 _vs. PC_7 _space. The PC_7 _high and low extreme gene sets are highlighted in red and blue colors, respectively. Click here for file

Additional File 5 The supplemental files provided with this publication are only a representative set of those generated by the PCA interpretation software. The complete collections of PCA interpretation results for both the GNF and diabetes datasets are provided as a supplement to this publication at [35].
Gene trajectory plots for PC_7 _high and low extreme gene sets with tissues in the order in which the original data were provided. Click here for file

Additional File 6 The supplemental files provided with this publication are only a representative set of those generated by the PCA interpretation software. The complete collections of PCA interpretation results for both the GNF and diabetes datasets are provided as a supplement to this publication at [35].
Gene trajectory plots for PC_7 _high and low extreme gene sets with tissues ordered by decreasing mean differences, and thus grouped by significance (up group at left, flat group in middle and low group at right). Click here for file

## Figures and Tables

**Figure 2:**
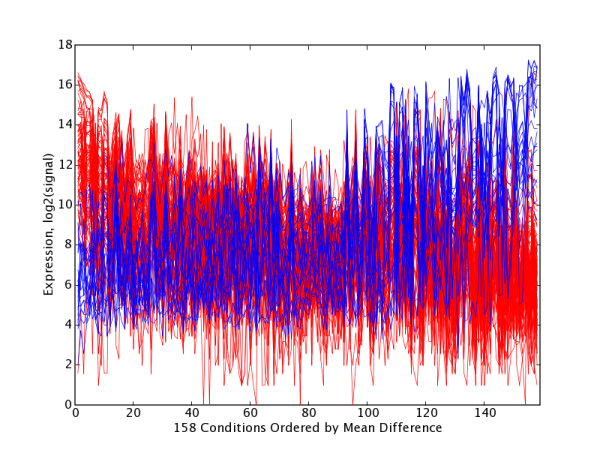
**GNF PC7 Extreme Gene Trajectory Plot with Tissues Ordered by Significance**. Trajectory plots for high and low extreme gene sets *H_7 _*(red, *n *= 88) and *L_7 _*(blue, *n *= 20) with tissues ordered by decreasing *mean*(*H_7_*) – *mean*(*L_7_*), and thus grouped by significance (*UP_7 _*group at left, *FLAT_7 _*group in middle and *LOW_7 _*group at right) at *test1Thresh *= 0.05. Table 3 lists the tissues within *UP_7 _*and *DOWN_7 _*that occur at each end of this plot.

**Figure 3:**
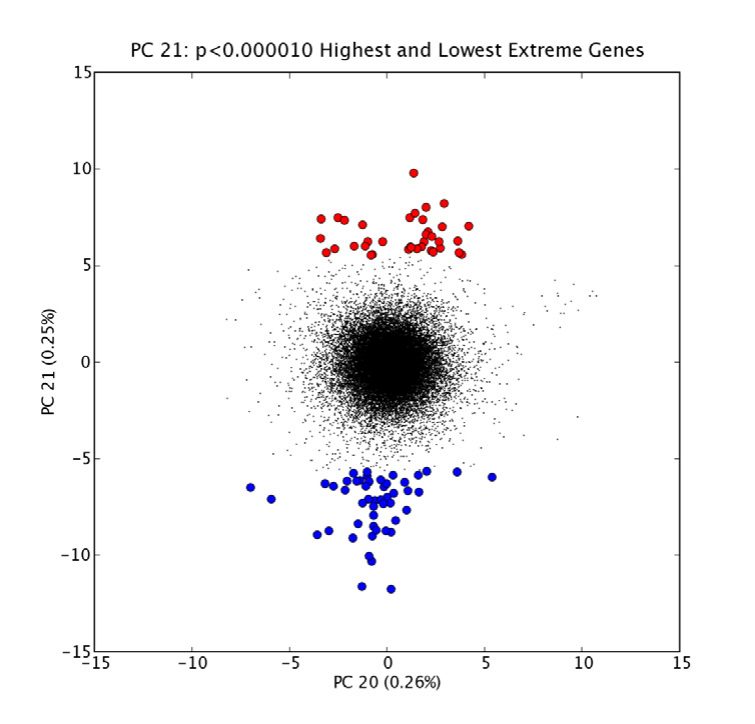
**GNF PC21 High and Low Extreme Gene Probes**. Scatter plot of N = 33689 probe expression levels projected onto PC_20 _vs. PC_21 _space with high and low extreme gene sets *H_21 _*(red points, *n *= 37) and *L_21 _*(blue points, *n *= 49); extreme genes selected at *extremeThresh *= 0.00001. The extreme genes in *L_21 _*are listed in Table 4.

**Figure 4:**
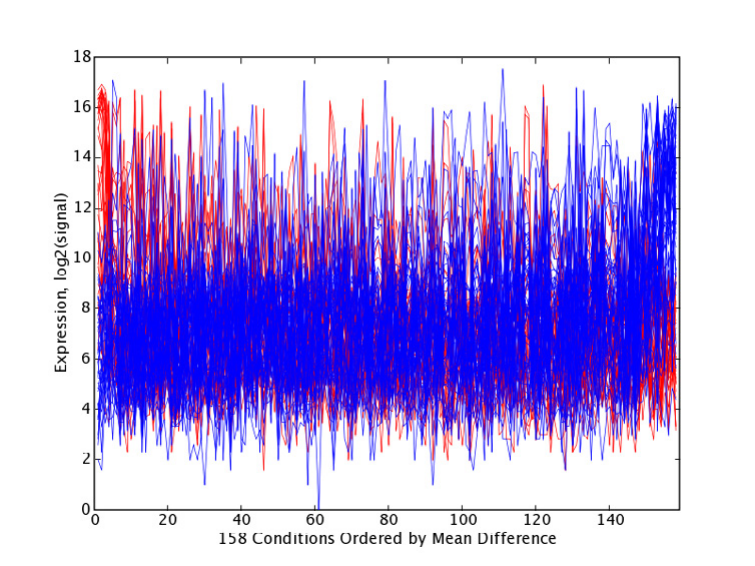
**GNF PC21 Extreme Gene Trajectory Plot with Tissues Ordered by Significance**. Trajectory plots for high and low extreme gene sets *H_21 _*(red, *n *= 37) and *L_21 _*(blue, *n *= 49) with tissues ordered by decreasing *mean*(*H_21_*) – *mean*(*L_21_*), and thus grouped by significance (*UP_21 _*group at left, *FLAT_21 _*group in middle and *LOW_21 _*group at right) at *test1Thresh *= 0.05. Table 5 lists the tissues within *UP_21 _*and *DOWN_21 _*that occur at each end of this plot.

**Table 1:** Variance Explained By Principal Components. Table 1 lists the percentage of variance in the GNF human tissue microarray data explained by each principal component. The first 10 components explain 80.36% of the total variance. Principal components 11 through 158 each explain less than 0.5% of the total variance, but combined explain almost 20%.

**Principal Component**	**Percentage of Variance**
1	67.62
2	4.66
3	2.04
4	1.41
5	1.07
6	0.90
7	0.81
8	0.69
9	0.63
10	0.54
11	0.46
12	0.42
13	0.37
14	0.35
15	0.32
16	0.31
17	0.30
18	0.29
19	0.26
20	0.26
21	0.25
22	0.24
23	0.23
24	0.22
25	0.22
26	0.22
27	0.21
28	0.20
29	0.20
30	0.19
31	0.19
32	0.19
33	0.18
34	0.18
35	0.18
36	0.18
37	0.17
38	0.16
39	0.16
40	0.16
41	0.16
42	0.16
43	0.16
44	0.15
45	0.15
46	0.15
47	0.15
48	0.15
49	0.14
50	0.14

**Table 2:** PC7 High Extreme Genes. Detailed info for (*n *= 88) extreme genes in PC_7 _set *H_7_*, ordered most extreme first, including common name, description, and associated Gene Ontology terms (provided with the GNF dataset). These extreme genes are shown as red points in Figure 1.

**PC-7 Value**	**Name**	**Description**	**Function**
18.63	*COL1A2*	collagen, type I, alpha 2	(GO:0006817) phosphate transport; (GO:0001501) skeletal development; (GO:0008147) structural constituent of bone;
17.89	*LUM*	lumican	(GO:0005518) collagen binding; (GO:0030199) collagen fibril organization; (GO:0005207) extracellular matrix glycoprotein; (GO:0005201) extracellular matrix structural constituent; (GO:0005203) proteoglycan; (GO:0007601) visual perception;
15.48	*TPM2*	tropomyosin 2 (beta)	(GO:0003779) actin binding; (GO:0007517) muscle development; (GO:0008307) structural constituent of muscle;
15.30	*IGFBP7*	insulin-like growth factor binding protein 7	(GO:0005520) insulin-like growth factor binding; (GO:0008285) negative regulation of cell proliferation; (GO:0001558) regulation of cell growth;
14.54	*CAV1*	caveolin 1, caveolae protein, 22 kDa	(GO:0005198) structural molecule activity; (GO:0008181) tumor suppressor;
14.08	*COL3A1*	collagen, type III, alpha 1 (Ehlers-Danlos syndrome type IV, autosomal dominant)	(GO:0008015) circulation; (GO:0005202) collagen; (GO:0005201) extracellular matrix structural constituent; (GO:0007397) histogenesis and organogenesis; (GO:0009887) organogenesis; (GO:0006817) phosphate transport;
13.84	*KCTD12*	potassium channel tetramerisation domain containing 12	(GO:0006813) potassium ion transport; (GO:0005515) protein binding; (GO:0005249) voltage-gated potassium channel activity;
13.83	*COL1A1*	collagen, type I, alpha 1	(GO:0005202) collagen; (GO:0005201) extracellular matrix structural constituent; (GO:0007605) perception of sound; (GO:0006817) phosphate transport; (GO:0001501) skeletal development; (GO:0008147) structural constituent of bone;
13.71	*MGP*	matrix Gla protein	(GO:0005509) calcium ion binding; (GO:0005201) extracellular matrix structural constituent; (GO:0007048) oncogenesis; (GO:0007605) perception of sound; (GO:0008147) structural constituent of bone;
13.57	*COL3A1*	collagen, type III, alpha 1 (Ehlers-Danlos syndrome type IV, autosomal dominant)	(GO:0008015) circulation; (GO:0005202) collagen; (GO:0005201) extracellular matrix structural constituent; (GO:0007397) histogenesis and organogenesis; (GO:0009887) organogenesis; (GO:0006817) phosphate transport;
13.25	*CALD1*	caldesmon 1	(GO:0003779) actin binding; (GO:0005516) calmodulin binding; (GO:0006936) muscle contraction; (GO:0007517) muscle development; (GO:0017022) myosin binding; (GO:0005523) tropomyosin binding
13.19	*MYL9*	myosin, light polypeptide 9, regulatory	(GO:0005509) calcium ion binding; (GO:0008307) structural constituent of muscle
13.12	*FN1*	fibronectin 1	(GO:0006953) acute-phase response; (GO:0007155) cell adhesion; (GO:0016477) cell migration; (GO:0005518) collagen binding; (GO:0005201) extracellular matrix structural constituent; (GO:0008201) heparin binding; (GO:0009611) response to wounding
12.71	*PLK2*	polo-like kinase 2 (Drosophila)	(GO:0005524) ATP binding; (GO:0043123) positive regulation of I-kappaB kinase/NF-kappaB cascade; (GO:0006468) protein amino acid phosphorylation; (GO:0004674) protein serine/threonine kinase activity; (GO:0004871) signal transducer activity; (GO:0016740) transferase activity
12.59	*CALD1*	caldesmon 1	(GO:0003779) actin binding; (GO:0005516) calmodulin binding; (GO:0006936) muscle contraction; (GO:0007517) muscle development; (GO:0017022) myosin binding; (GO:0005523) tropomyosin binding
12.54	*CTGF*	connective tissue growth factor	(GO:0006259) DNA metabolism; (GO:0007155) cell adhesion; (GO:0005194) cell adhesion molecule activity; (GO:0008151) cell growth and/or maintenance; (GO:0006928) cell motility; (GO:0008201) heparin binding; (GO:0005520) insulin-like growth factor binding; (GO:0005515) protein binding; (GO:0001558) regulation of cell growth; (GO:0009611) response to wounding
12.39	*D2S448*	Melanoma associated gene	(GO:0006955) immune response; (GO:0005152) interleukin-1 receptor antagonist activity; (GO:0004601) peroxidase activity
12.28	*MFAP5*	microfibrillar associated protein 5	(GO:0005201) extracellular matrix structural constituent
12.24	*SEMA3C*	sema domain, immunoglobulin domain (Ig), short basic domain, secreted, (semaphorin) 3C	(GO:0008151) cell growth and/or maintenance; (GO:0007275) development; (GO:0009315) drug resistance; (GO:0006955) immune response; (GO:0042493) response to drug
12.20	*ADAMTS1*	a disintegrin-like and metalloprotease (reprolysin type) with thrombospondin type 1 motif, 1	(GO:0008201) heparin binding; (GO:0016787) hydrolase activity; (GO:0005178) integrin binding; (GO:0007229) integrin-mediated signaling pathway; (GO:0004222) metalloendopeptidase activity; (GO:0008237) metallopeptidase activity; (GO:0008285) negative regulation of cell proliferation; (GO:0006508) proteolysis and peptidolysis; (GO:0008270) zinc ion binding
11.99	*SPARC*	secreted protein, acidic, cysteine-rich (osteonectin)	(GO:0005509) calcium ion binding; (GO:0005518) collagen binding
11.76	*F3*	coagulation factor III (thromboplastin, tissue factor)	(GO:0007596) blood coagulation; (GO:0003801) blood coagulation factor activity; (GO:0004896) hematopoietin/interferon-class (D200-domain) cytokine receptor activity; (GO:0006955) immune response; (GO:0004872) receptor activity; (GO:0004888) transmembrane receptor activity
11.71	*PLS3*	plastin 3 (T isoform)	(GO:0003779) actin binding; (GO:0005509) calcium ion binding
11.70	*THBS1*	thrombospondin 1	(GO:0007596) blood coagulation; (GO:0005509) calcium ion binding; (GO:0007155) cell adhesion; (GO:0005194) cell adhesion molecule activity; (GO:0006928) cell motility; (GO:0007275) development; (GO:0004866) endopeptidase inhibitor activity; (GO:0008201) heparin binding; (GO:0007399) neurogenesis; (GO:0005515) protein binding; (GO:0004871) signal transducer activity; (GO:0005198) structural molecule activity
11.69	*PLOD2*	procollagen-lysine, 2-oxoglutarate 5-dioxygenase (lysine hydroxylase) 2	(GO:0016491) oxidoreductase activity; (GO:0016702) oxidoreductase activity\, acting on single donors with incorporation of molecular oxygen\, incorporation of two atoms of oxygen; (GO:0008475) procollagen-lysine 5-dioxygenase activity; (GO:0019538) protein metabolism; (GO:0006464) protein modification
11.37	*CAV1*	caveolin 1, caveolae protein, 22 kDa	(GO:0005198) structural molecule activity; (GO:0008181) tumor suppressor
11.36	*gnf1h04130_x_at*	None	None
11.28	*FN1*	fibronectin 1	(GO:0006953) acute-phase response; (GO:0007155) cell adhesion; (GO:0016477) cell migration; (GO:0005518) collagen binding; (GO:0005201) extracellular matrix structural constituent; (GO:0008201) heparin binding; (GO:0009611) response to wounding
11.18	*SMARCA1*	SWI/SNF related, matrix associated, actin dependent regulator of chromatin, subfamily a, member 1	(GO:0005524) ATP binding; (GO:0008026) ATP-dependent helicase activity; (GO:0003677) DNA binding; (GO:0004002) adenosinetriphosphatase; (GO:0006338) chromatin remodeling; (GO:0004386) helicase activity; (GO:0045182) translation regulator activity
11.08	*TAGLN*	transgelin	(GO:0007517) Muscle development
10.95	*COL4A1*	collagen, type IV, alpha 1	(GO:0003677) DNA binding; (GO:0005202) collagen; (GO:0005201) extracellular matrix structural constituent; (GO:0006817) phosphate transport
10.90	*CAV2*	caveolin 2	(GO:0008181) tumor suppressor
10.90	*TPM1*	tropomyosin 1 (alpha)	(GO:0003779) actin binding; (GO:0007517) muscle development; (GO:0008016) regulation of heart rate; (GO:0005200) structural constituent of cytoskeleton; (GO:0008307) structural constituent of muscle
10.85	*GJA1*	gap junction protein, alpha 1, 43 kDa (connexin 43)	(GO:0007267) cell-cell signaling; (GO:0015285) connexon channel activity; (GO:0007507) heart development; (GO:0015075) ion transporter activity; (GO:0006936) muscle contraction; (GO:0007605) perception of sound; (GO:0043123) positive regulation of I-kappaB kinase/NF-kappaB cascade; (GO:0004871) signal transducer activity; (GO:0006832) small molecule transport; (GO:0006810) transport
10.82	*COL1A2*	collagen, type I, alpha 2	(GO:0005202) collagen; (GO:0005201) extracellular matrix structural constituent; (GO:0006817) phosphate transport; (GO:0001501) skeletal development; (GO:0008147) structural constituent of bone
10.61	*FXR1*	fragile X mental retardation, autosomal homolog 1	(GO:0003677) DNA binding; (GO:0003723) RNA binding; (GO:0006915) apoptosis; (GO:0003676) nucleic acid binding; (GO:0006913) nucleocytoplasmic transport
10.60	*COL6A3*	collagen, type VI, alpha 3	cell adhesion (GO:0007155);cell adhesion molecule activity (GO:0005194);collagen (GO:0005202);extracellular matrix structural constituent (GO:0005201);muscle development (GO:0007517);phosphate transport (GO:0006817);protein binding (GO:0005515);serine-type endopeptidase inhibitor activity (GO:0004867)
10.52	*CYR61*	cysteine-rich, angiogenic inducer, 61	cell adhesion (GO:0007155);cell proliferation (GO:0008283);chemotaxis (GO:0006935);embryogenesis and morphogenesis (GO:0007345);heparin binding (GO:0008201);insulin-like growth factor binding (GO:0005520);morphogenesis (GO:0009653);regulation of cell growth (GO:0001558)
10.50	*10-Sep*	septin 10	GTP binding (GO:0005525)
10.44	*IL1R1*	interleukin 1 receptor, type I	cell surface receptor linked signal transduction (GO:0007166);immune response (GO:0006955);inflammatory response (GO:0006954);interleukin-1 receptor activity (GO:0004908);interleukin-1\, Type I\, activating receptor activity (GO:0004909);signal transducer activity (GO:0004871);transmembrane receptor activity (GO:0004888)
10.37	*WBP5*	WW domain binding protein 1	DNA binding (GO:0003677)
10.35	*LAMB1*	laminin, beta 1	cell adhesion (GO:0007155);cell adhesion molecule activity (GO:0005194);protein binding (GO:0005515);structural molecule activity (GO:0005198)
10.33	*EMP1*	epithelial membrane protein 1	cell death (GO:0008219);cell growth (GO:0016049);cell proliferation (GO:0008283);development (GO:0007275);oncogenesis (GO:0007048)
10.23	*NRP1*	neuropilin 1	axon guidance (GO:0007411);cell adhesion (GO:0007155);cell-cell signaling (GO:0007267);histogenesis and organogenesis (GO:0007397);neurogenesis (GO:0007399);organogenesis (GO:0009887);positive regulation of cell proliferation (GO:0008284);receptor activity (GO:0004872);signal transduction (GO:0007165);vascular endothelial growth factor receptor activity (GO:0005021)
10.12	*COL3A1*	collagen, type III, alpha 1 (Ehlers-Danlos syndrome type IV, autosomal dominant)	circulation (GO:0008015);collagen (GO:0005202);extracellular matrix structural constituent (GO:0005201);histogenesis and organogenesis (GO:0007397);organogenesis (GO:0009887);phosphate transport (GO:0006817)
10.10	*CALD1*	caldesmon 1	actin binding (GO:0003779);calmodulin binding (GO:0005516);muscle contraction (GO:0006936);muscle development (GO:0007517);myosin binding (GO:0017022);tropomyosin binding (GO:0005523)
10.10	*THBS1*	thrombospondin 1	blood coagulation (GO:0007596);calcium ion binding (GO:0005509);cell adhesion (GO:0007155);cell adhesion molecule activity (GO:0005194);cell motility (GO:0006928);development (GO:0007275);endopeptidase inhibitor activity (GO:0004866);heparin binding (GO:0008201);neurogenesis (GO:0007399);protein binding (GO:0005515);signal transducer activity (GO:0004871);structural molecule activity (GO:0005198)
10.05	*FN1*	fibronectin 1	acute-phase response (GO:0006953);cell adhesion (GO:0007155);cell migration (GO:0016477);collagen binding (GO:0005518);extracellular matrix structural constituent (GO:0005201);heparin binding (GO:0008201);response to wounding (GO:0009611)
10.03	*SPARC*	secreted protein, acidic, cysteine-rich (osteonectin)	calcium ion binding (GO:0005509);collagen binding (GO:0005518)
10.02	*IGFBP7*	insulin-like growth factor binding protein 7	insulin-like growth factor binding (GO:0005520);negative regulation of cell proliferation (GO:0008285);regulation of cell growth (GO:0001558)
9.99	*IL6ST*	interleukin 6 signal transducer (gp130, oncostatin M receptor)	cell surface receptor linked signal transduction (GO:0007166);gp130 (GO:0004898);immune response (GO:0006955);interleukin-6 receptor activity (GO:0004915);oncostatin-M receptor activity (GO:0004924);receptor activity (GO:0004872);signal transduction (GO:0007165)
9.98	*LAMB1*	laminin, beta 1	cell adhesion (GO:0007155);cell adhesion molecule activity (GO:0005194);protein binding (GO:0005515);structural molecule activity (GO:0005198)
9.94	*TAZ*	transcriptional co-activator with PDZ-binding motif (TAZ)	transcription coactivator activity (GO:0003713)
9.93	*DCN*	decorin	chondroitin sulfate/dermatan sulfate proteoglycan (GO:0005205);histogenesis and organogenesis (GO:0007397);organogenesis (GO:0009887)
9.93	*ANXA1*	annexin A1	calcium ion binding (GO:0005509);calcium-dependent phospholipid binding (GO:0005544);cell motility (GO:0006928);cell surface receptor linked signal transduction (GO:0007166);inflammatory response (GO:0006954);lipid metabolism (GO:0006629);phospholipase A2 inhibitor activity (GO:0019834);phospholipase inhibitor activity (GO:0004859);phospholipid binding (GO:0005543);receptor binding (GO:0005102)
9.89	*MYH11*	myosin, heavy polypeptide 11, smooth muscle	ATP binding (GO:0005524);actin binding (GO:0003779);calmodulin binding (GO:0005516);cell growth and/or maintenance (GO:0008151);motor activity (GO:0003774);muscle development (GO:0007517);protein amino acid alkylation (GO:0008213);striated muscle contraction (GO:0006941)
9.89	*EFEMP1*	EGF-containing fibulin-like extracellular matrix protein 1	calcium ion binding (GO:0005509);visual perception (GO:0007601)
9.88	*SPUVE*	protease, serine, 23	chymotrypsin activity (GO:0004263);hydrolase activity (GO:0016787);proteolysis and peptidolysis (GO:0006508);trypsin activity (GO:0004295)
9.87	*Hs.514018*	CDNA: FLJ22209 fis, clone HRC01496	None
9.80	*FN1*	fibronectin 1	acute-phase response (GO:0006953);cell adhesion (GO:0007155);cell migration (GO:0016477);collagen binding (GO:0005518);extracellular matrix structural constituent (GO:0005201);heparin binding (GO:0008201);response to wounding (GO:0009611)
9.77	*COL16A1*	collagen, type XVI, alpha 1	cell adhesion (GO:0007155);collagen (GO:0005202);extracellular matrix structural constituent (GO:0005201);phosphate transport (GO:0006817);pregnancy (GO:0007565)
9.73	*SNX7*	sorting nexin 7	intracellular protein transport (GO:0006886);intracellular signaling cascade (GO:0007242);protein transporter activity (GO:0008565)
9.69	*AHR*	aryl hydrocarbon receptor	DNA binding (GO:0003677);apoptosis (GO:0006915);ligand-dependent nuclear receptor activity (GO:0004879);response to stress (GO:0006950);response to xenobiotic stimulus (GO:0009410);signal transduction (GO:0007165);transcription factor activity (GO:0003700);transcription from Pol II promoter (GO:0006366)
9.57	*COL6A1*	collagen, type VI, alpha 1	DNA binding (GO:0003677);cell adhesion (GO:0007155);cell adhesion molecule activity (GO:0005194);collagen (GO:0005202);extracellular matrix structural constituent (GO:0005201);histogenesis and organogenesis (GO:0007397);molecular_function unknown (GO:0005554);phosphate transport (GO:0006817);protein binding (GO:0005515)
9.55	*KIAA0992*	palladin	amino acid metabolism (GO:0006520)
9.54	*COL5A2*	collagen, type V, alpha 2	cell growth and/or maintenance (GO:0008151);collagen (GO:0005202);extracellular matrix glycoprotein (GO:0005207);extracellular matrix structural constituent (GO:0005201);phosphate transport (GO:0006817)
9.53	*FBN1*	fibrillin 1 (Marfan syndrome)	calcium ion binding (GO:0005509);development (GO:0007275);extracellular matrix structural constituent (GO:0005201);skeletal development (GO:0001501);visual perception (GO:0007601)
9.51	*PAM*	peptidylglycine alpha-amidating monooxygenase	electron transporter activity (GO:0005489);monooxygenase activity (GO:0004497);peptide amidation (GO:0001519);peptidylglycine monooxygenase activity (GO:0004504);protein modification (GO:0006464)
9.50	*LOC92912*	hypothetical protein LOC92912	ligase activity (GO:0016874);ubiquitin conjugating enzyme activity (GO:0004840);ubiquitin cycle (GO:0006512);ubiquitin-protein ligase activity (GO:0004842)
9.47	*COL6A2*	collagen, type VI, alpha 2	cell-cell adhesion (GO:0016337);collagen (GO:0005202);extracellular matrix organization and biogenesis (GO:0030198);extracellular matrix structural constituent (GO:0005201);muscle development (GO:0007517);phosphate transport (GO:0006817);protein binding, bridging (GO:0030674)
9.45	*PTX1*	PTX1 protein	None
9.41	*KIAA0992*	palladin	amino acid metabolism (GO:0006520)
9.33	*CSPG2*	chondroitin sulfate proteoglycan 2 (versican)	calcium ion binding (GO:0005509);cell adhesion (GO:0007155);chondroitin sulfate proteoglycan (GO:0005204);development (GO:0007275);glycosaminoglycan binding (GO:0005539);heterophilic cell adhesion (GO:0007157);hyaluronic acid binding (GO:0005540);lectin (GO:0005530);proteoglycan (GO:0005203);sugar binding (GO:0005529)
9.32	*CSPG2*	chondroitin sulfate proteoglycan 2 (versican)	calcium ion binding (GO:0005509);cell adhesion (GO:0007155);chondroitin sulfate proteoglycan (GO:0005204);development (GO:0007275);glycosaminoglycan binding (GO:0005539);heterophilic cell adhesion (GO:0007157);hyaluronic acid binding (GO:0005540);lectin (GO:0005530);proteoglycan (GO:0005203);sugar binding (GO:0005529)
9.30	*SERPINH1*	serine (or cysteine) proteinase inhibitor, clade H (heat shock protein 47), member 1, (collagen binding protein 1)	collagen binding (GO:0005518);heat shock protein activity (GO:0003773);response to stress (GO:0006950);serine-type endopeptidase inhibitor activity (GO:0004867);serpin (GO:0004868)
9.23	*TM4SF6*	transmembrane 4 superfamily member 6	cell adhesion molecule activity (GO:0005194);cell motility (GO:0006928);positive regulation of I-kappaB kinase/NF-kappaB cascade (GO:0043123);signal transducer activity (GO:0004871)
9.20	*PTRF*	polymerase I and transcript release factor	None
9.19	*LATS2*	LATS, large tumor suppressor, homolog 2 (Drosophila)	ATP binding (GO:0005524);protein amino acid phosphorylation (GO:0006468);protein serine/threonine kinase activity (GO:0004674);transferase activity (GO:0016740);tumor suppressor (GO:0008181)
9.12	*CTBP2*	C-terminal binding protein 2	L-serine biosynthesis (GO:0006564);negative regulation of cell proliferation (GO:0008285);oxidoreductase activity (GO:0016491);oxidoreductase activity, acting on the CH-OH group of donors, NAD or NADP as acceptor (GO:0016616);tumor suppressor (GO:0008181);viral replication (GO:0008166)
9.06	*ITGAV*	integrin, alpha V (vitronectin receptor, alpha polypeptide, antigen CD51)	cell adhesion (GO:0007155);cell adhesion receptor activity (GO:0004895);cell-matrix adhesion (GO:0007160);integrin-mediated signaling pathway (GO:0007229);protein binding (GO:0005515)
9.05	*RCN2*	reticulocalbin 2, EF-hand calcium binding domain	calcium ion binding (GO:0005509);protein binding (GO:0005515);tumor suppressor (GO:0008181)
8.98	*CPD*	carboxypeptidase D	carboxypeptidase A activity (GO:0004182);carboxypeptidase D activity (GO:0004187);carboxypeptidase activity (GO:0004180);hydrolase activity (GO:0016787);proteolysis and peptidolysis (GO:0006508);zinc ion binding (GO:0008270)
8.98	*FLJ21174*	hypothetical protein FLJ21174	None
8.96	*MGC5395*	hypothetical protein MGC5395	intracellular signaling cascade (GO:0007242);neurogenesis (GO:0007399);protein binding (GO:0005515)
8.96	*MFAP2*	microfibrillar-associated protein 2	extracellular matrix glycoprotein (GO:0005207);extracellular matrix structural constituent (GO:0005201)
8.94	*CRIM1*	cysteine-rich motor neuron 1	cysteine-type endopeptidase activity (GO:0004197);insulin-like growth factor binding (GO:0005520);insulin-like growth factor receptor activity (GO:0005010);neurogenesis (GO:0007399);proteolysis and peptidolysis (GO:0006508);regulation of cell growth (GO:0001558);serine-type endopeptidase inhibitor activity (GO:0004867)
8.92	*ALDH1A3*	aldehyde dehydrogenase 1 family, member A3	alcohol metabolism (GO:0006066);aldehyde dehydrogenase [NAD(P)+] activity (GO:0004030);aldehyde dehydrogenase activity (GO:0004028);lipid metabolism (GO:0006629);metabolism (GO:0008152);oxidoreductase activity (GO:0016491)
8.92	*MGC34132*	hypothetical protein MGC34132	None

**Table 3:** PC7 Tissue Partitioning and Ordering. Partitioning and ordering of tissues into sets *UP_7_*(*n *= 51) and *DOWN_7_*(*n *= 48) sets found to have significant expression differences for *H_7 _*and *L_7 _*at *test1Thresh *= 0.05. Tissues within groups are ordered by decreasing abs (*mean *(*H_7_*)–*mean*(*L_7_*)), which has the effect of placing the most significantly affected tissues at the top of each list. The most significant tissues in *UP_7 _*are at the left of Figure 2, and the most significant conditions in *DOWN_7 _*are at the right of Figure 2.

**UP7**	**DOWN7**
SmoothMuscle	PB-CD19+Bcells
SmoothMuscle	PB-CD19+Bcells
CardiacMyocytes	PB-BDCA4+Dentritic_Cells
ADIPOCYTE	PB-BDCA4+Dentritic_Cells
ADIPOCYTE	lymphomaburkittsRaji
CardiacMyocytes	lymphomaburkittsDaudi
TestisGermCell	lymphomaburkittsDaudi
bronchialepithelialcells	lymphomaburkittsRaji
bronchialepithelialcells	PB-CD56+NKCells
PLACENTA	bonemarrow
PLACENTA	bonemarrow
TestisInterstitial	PB-CD56+NKCells
UterusCorpus	leukemiapromyelocytic(hl60)
Uterus	721_B_lymphoblasts
FetalThyroid	Tonsil
OlfactoryBulb	Tonsil
TestisLeydigCell	WHOLEBLOOD
UterusCorpus	721_B_lymphoblasts
FetalThyroid	leukemiapromyelocytic(hl60)
atrioventricularnode	WHOLEBLOOD
TestisInterstitial	thymus
TestisLeydigCell	PB-CD8+Tcells
TestisSeminiferousTubule	PB-CD8+Tcells
atrioventricularnode	thymus
DRG	lymphnode
Fetallung	Heart
Uterus	lymphnode
Ciliaryganglion	PB-CD14+Monocytes
TestisGermCell	PB-CD4+Tcells
AdrenalCortex	PB-CD4+Tcells
Fetallung	PB-CD14+Monocytes
Ciliaryganglion	BM-CD71+EarlyErythroid
DRG	BM-CD34+
TONGUE	Liver
SuperiorCervicalGanglion	Heart
Ovary	BM-CD71+EarlyErythroid
TestisSeminiferousTubule	BM-CD34+
Skin	BM-CD105+Endothelial
OlfactoryBulb	salivarygland
TONGUE	leukemialymphoblastic(molt4)
Ovary	Liver
TrigeminalGanglion	Lung
AdrenalCortex	Lung
PancreaticIslets	leukemialymphoblastic(molt4)
Skin	BM-CD105+Endothelial
Fetalbrain	BM-CD33+Myeloid
TrigeminalGanglion	salivarygland
Fetalbrain	BM-CD33+Myeloid
Amygdala	
SuperiorCervicalGanglion	
PrefrontalCortex	

**Table 4:** PC21 Low Extreme Genes. Detailed info for (*n *= 49) extreme genes in PC_21 _set *L_21_*, ordered most extreme first, including common name, description, and associated Gene Ontology terms (provided with the GNF dataset). These extreme genes are shown as blue points in Figure 3.

**PC-21 Value**	***Name***	**Description**	**Function**
-11.74	*Ckm*	creatine kinase, muscle	creatine kinase activity (GO:0004111); transferase activity, transferring phosphorus-containing groups (GO:0016772)
-11.61	*ACTA1*	actin, alpha 1, skeletal muscle	motor activity (GO:0003774);muscle contraction (GO:0006936);muscle development (GO:0007517);structural constituent of cytoskeleton (GO:0005200)
-10.27	*MYH7*	myosin, heavy polypeptide 7, cardiac muscle, beta	ATP binding (GO:0005524);actin binding (GO:0003779);calmodulin binding (GO:0005516);microfilament motor activity (GO:0000146);motor activity (GO:0003774);muscle contraction (GO:0006936);muscle development (GO:0007517);protein amino acid alkylation (GO:0008213);striated muscle contraction (GO:0006941);structural constituent of muscle (GO:0008307)
-10.01	*TPM1*	tropomyosin 1 (alpha)	actin binding (GO:0003779);muscle development (GO:0007517);regulation of heart rate (GO:0008016);structural constituent of cytoskeleton (GO:0005200);structural constituent of muscle (GO:0008307)
-9.10	*MYL1*	myosin, light polypeptide 1, alkali; skeletal, fast	calcium ion binding (GO:0005509);muscle development (GO:0007517);structural constituent of muscle (GO:0008307)
-9.10	*TNNC1*	troponin C, slow	calcium ion binding (GO:0005509);muscle development (GO:0007517)
-8.92	*PPP1R1A*	protein phosphatase 1, regulatory (inhibitor) subunit 1A	glycogen metabolism (GO:0005977);protein phosphatase inhibitor activity (GO:0004864);signal transduction (GO:0007165);type 1 serine/threonine specific protein phosphatase inhibitor activity (GO:0004865)
-8.79	*TNNC2*	troponin C2, fast	calcium ion binding (GO:0005509);muscle development (GO:0007517)
-8.71	*TNNT1*	troponin T1, skeletal, slow	muscle development (GO:0007517);tropomyosin binding (GO:0005523)
-8.70	*KRT14*	keratin 14 (epidermolysis bullosa simplex, Dowling-Meara, Koebner)	biological_process unknown (GO:0000004);structural constituent of cytoskeleton (GO:0005200);structural constituent of epidermis (GO:0030280)
-8.67	*TTID*	titin immunoglobulin domain protein (myotilin)	muscle contraction (GO:0006936);protein binding- (GO:0005515);structural constituent of muscle (GO:0008307)
-8.49	*HUMMLC2B*	myosin light chain 2	calcium ion binding (GO:0005509);structural constituent of muscle (GO:0008307)
-8.35	*TRIM*	T-cell receptor interacting molecule	DNA binding (GO:0003677);cellular defense response (GO:0006968);signal transduction (GO:0007165);transmembrane receptor protein tyrosine kinase adaptor protein activity (GO:0005068)
-8.20	*TTN*	titin	ATP binding (GO:0005524);calmodulin binding (GO:0005516);cation transporter activity (GO:0008324);hematopoietin/interferon-class (D200-domain) cytokine receptor activity (GO:0004896);muscle development (GO:0007517);myosin binding (GO:0017022);protein amino acid phosphorylation (GO:0006468);protein serine/threonine kinase activity (GO:0004674);regulation of actin filament length (GO:0030832);somatic muscle development (GO:0007525);striated muscle contraction (GO:0006941);structural constituent of muscle (GO:0008307);structural molecule activity (GO:0005198);transferase activity (GO:0016740)
-7.91	*CSRP3*	cysteine and glycine-rich protein 3 (cardiac LIM protein)	None
-7.66	*MB*	myoglobin	electron transporter activity (GO:0005489);globin (GO:0001524);oxygen transport (GO:0015671);oxygen transporter activity (GO:0005344);transport (GO:0006810)
-7.46	*ENO3*	enolase 3, (beta, muscle)	glycolysis (GO:0006096);lyase activity (GO:0016829);magnesium ion binding (GO:0000287);phosphopyruvate hydratase activity (GO:0004634)
-7.32	*NTRK2*	neurotrophic tyrosine kinase, receptor, type 2	ATP binding (GO:0005524);kinase activity (GO:0016301);neurogenesis (GO:0007399);neurotrophin TRKB receptor activity (GO:0005015);neurotrophin binding (GO:0043121);protein amino acid phosphorylation (GO:0006468);receptor activity (GO:0004872);transferase activity (GO:0016740);transmembrane receptor protein tyrosine kinase activity (GO:0004714);transmembrane receptor protein tyrosine kinase signaling pathway (GO:0007169)
-7.28	*MYL2*	myosin, light polypeptide 2, regulatory, cardiac, slow	calcium ion binding (GO:0005509);muscle development (GO:0007517);structural constituent of muscle (GO:0008307)
-7.27	*TF*	Transferring	ferric iron binding (GO:0008199);iron ion binding (GO:0005506);iron ion homeostasis (GO:0006879);iron ion transport (GO:0006826);transport (GO:0006810)
-7.16	*ACSL1*	acyl-CoA synthetase long-chain family member 1	digestion (GO:0007586);fatty acid metabolism (GO:0006631);ligase activity (GO:0016874);long-chain-fatty-acid-CoA ligase activity (GO:0004467);magnesium ion binding (GO:0000287);metabolism (GO:0008152)
-7.10	*MYBPC1*	myosin binding protein C, slow type	actin binding (GO:0003779);cell adhesion (GO:0007155);cell adhesion molecule activity (GO:0005194);muscle development (GO:0007517);protein binding (GO:0005515);striated muscle contraction (GO:0006941);structural constituent of muscle (GO:0008307)
-7.07	*S100A2*	S100 calcium binding protein A2	biological_process unknown (GO:0000004);calcium ion binding (GO:0005509)
-7.07	*PYGM*	phosphorylase, glycogen; muscle (McArdle syndrome, glycogen storage disease type V)	amino acid metabolism (GO:0006520);carbohydrate metabolism (GO:0005975);glycogen metabolism (GO:0005977);glycogen phosphorylase activity (GO:0008184);transferase activity, transferring glycosyl groups (GO:0016757)
-6.99	*ACTN2*	actinin, alpha 2	actin binding (GO:0003779);calcium ion binding (GO:0005509);protein binding (GO:0005515);structural constituent of muscle (GO:0008307)
-6.76	*MYBPC2*	myosin binding protein C, fast type	actin binding (GO:0003779);cell adhesion (GO:0007155);cell adhesion molecule activity (GO:0005194);muscle development (GO:0007517);protein binding (GO:0005515);striated muscle contraction (GO:0006941);structural constituent of muscle (GO:0008307)
-6.71	*UCP2*	uncoupling protein 2 (mitochondrial, proton carrier)	binding (GO:0005488);mitochondrial transport (GO:0006839);proton transport (GO:0015992);small molecule transport (GO:0006832);transport (GO:0006810);transporter activity (GO:0005215);uncoupling protein activity (GO:0015302)
-6.63	*NEB*	nebulin	actin binding (GO:0003779);muscle development (GO:0007517);regulation of actin filament length (GO:0030832);somatic muscle development (GO:0007525);structural constituent of muscle (GO:0008307)
-6.60	*IDH2*	isocitrate dehydrogenase 2 (NADP+), mitochondrial	carbohydrate metabolism (GO:0005975);glyoxylate cycle (GO:0006097);isocitrate dehydrogenase (NADP+) activity (GO:0004450);main pathways of carbohydrate metabolism (GO:0006092);metabolism (GO:0008152);oxidoreductase activity (GO:0016491);tricarboxylic acid cycle (GO:0006099)
-6.47	*CSTA*	cystatin A (stefin A)	cysteine protease inhibitor activity (GO:0004869);endopeptidase inhibitor activity (GO:0004866)
-6.45	*MYH2*	myosin, heavy polypeptide 2, skeletal muscle, adult	ATP binding (GO:0005524);actin binding (GO:0003779);calmodulin binding (GO:0005516);microfilament motor activity (GO:0000146);motor activity (GO:0003774);muscle contraction (GO:0006936);muscle development (GO:0007517);muscle motor activity (GO:0003776);protein amino acid alkylation (GO:0008213);striated muscle contraction (GO:0006941)
-6.42	*KRT4*	keratin 4	cytoskeleton organization and biogenesis (GO:0007010);structural constituent of cytoskeleton (GO:0005200);structural molecule activity (GO:0005198)
-6.40	*CLIC6*	chloride intracellular channel 6	chloride transport (GO:0006821);ion transport (GO:0006811);voltage-gated chloride channel activity (GO:0005247)
-6.27	*SPRR1A*	small proline-rich protein 1A	structural molecule activity (GO:0005198)
-6.25	*TNNI2*	troponin I, skeletal, fast	actin binding (GO:0003779);muscle development (GO:0007517)
-6.22	*TNNI1*	troponin I, skeletal, slow	actin binding (GO:0003779);muscle development (GO:0007517);tropomyosin binding (GO:0005523)
-6.17	*LDB3*	LIM domain binding 3	electron transport (GO:0006118);electron transporter activity (GO:0005489);protein binding (GO:0005515)
-6.14	*CRYAB*	crystallin, alpha B	chaperone activity (GO:0003754);muscle contraction (GO:0006936);protein folding (GO:0006457);structural constituent of eye lens (GO:0005212);visual perception (GO:0007601)
-6.12	*HFL1*	H factor (complement)-like 1	plasma protein (GO:0005209)
-6.15	*S100A1*	S100 calcium binding protein A1	calcium ion binding (GO:0005509);cell communication (GO:0007154);intracellular signaling cascade (GO:0007242);neurogenesis (GO:0007399);protein binding (GO:0005515);zinc ion binding (GO:0008270)
-6.09	*HSPB8*	heat shock 22 kDa protein 8	biological_process unknown (GO:0000004);heat shock protein activity (GO:0003773);protein serine/threonine kinase activity (GO:0004674);transferase activity (GO:0016740)
-5.93	*PPFIBP2*	PTPRF interacting protein, binding protein 2 (liprin beta 2)	None
-5.88	*GHR*	growth hormone receptor	endocytosis (GO:0006897);growth hormone receptor activity (GO:0004903);growth pattern (GO:0007150);receptor activity (GO:0004872);skeletal development (GO:0001501)
-5.85	*ACSL1*	acyl-CoA synthetase long-chain family member 1	digestion (GO:0007586);fatty acid metabolism (GO:0006631);ligase activity (GO:0016874);long-chain-fatty-acid-CoA ligase activity (GO:0004467);magnesium ion binding (GO:0000287);metabolism (GO:0008152)
-5.84	*MGC42493*	hypothetical protein MGC42493	nucleic acid binding (GO:0003676);zinc ion binding (GO:0008270)
-5.73	*SCD*	stearoyl-CoA desaturase (delta-9-desaturase)	fatty acid biosynthesis (GO:0006633);iron ion binding (GO:0005506);oxidoreductase activity (GO:0016491);stearoyl-CoA 9-desaturase activity (GO:0004768)
-5.67	*hIAN2*	human immune associated nucleotide 2	GTP binding (GO:0005525)
-5.65	*PDE4DIP*	phosphodiesterase 4D interacting protein (myomegalin)	None
-5.63	*HBA2*	hemoglobin, alpha 2	oxygen transport (GO:0015671);oxygen transporter activity (GO:0005344);protein binding (GO:0005515);transport (GO:0006810)

**Table 5:** PC21 Tissue Partitioning and Ordering. Partitioning and ordering of tissues into sets *UP_21_*(*n *= 8) and *DOWN_21 _*(*n *= 20) sets found to have significant expression differences for *H_21 _*and *L_21 _*at *test1Thresh *= 0.05. Tissues within groups are ordered by decreasing abs(*mean *(*H_21_*)–*mean*(*L_21_*)), which has the effect of placing the most significantly affected tissues at the top of each list. The most significant tissues in *UP_21 _*are at the left of Figure 4, and the most significant conditions in *DOWN_21 _*are at the right of Figure 4.

**UP21**	**DOWN21**
Pancreas	TONGUE
Pancreas	SkeletalMuscle
PancreaticIslets	TONGUE
PancreaticIslets	SkeletalMuscle
Bonemarrow	Thyroid
Bonemarrow	Heart
BM-CD34+	Thyroid
BM-CD34+	FetalThyroid
	Heart
	FetalThyroid
	Testis
	721_B_lymphoblasts
	Testis
	ADIPOCYTE
	721_B_lymphoblasts
	ADIPOCYTE
	Thalamus
	Caudatenucleus
	Spinalcord
	Prostate

**Table 6:** GNF human expression principal components having extreme gene sets enriched in GO categories. Each of the GNF human expression PCEG sets, derived using an *extremeThresh *of 0.00001, was tested for Gene Ontology (GO) statistical enrichment. Terms that were enriched for a particular PC at 1% significance threshold are reported. 26 of the top 42 principal components produced extreme gene lists having significant GO term enrichment.

**PCEG Set**	**GO Description (p-value)**
01 low	GO:0006412 protein biosynthesis (4.51e-07)
03 high	GO:0005201 extracellular matrix structural constituent (1.43e-08)
03 low	GO:0007399 neurogenesis (5.05e-09) GO:0007268 synaptic transmission (9.6e-09) GO:0007417 central nervous system development (1.31e-08) GO:0016820 hydrolase activity, acting on acid anhydrides, catalyzing transmembrane movement of substances (4.85e-07)
04 low	GO:0005201 extracellular matrix structural constituent (5.82e-07)
05 high	GO:0007067 mitosis (3.43e-17) GO:0051301 cell division (4.45e-17) GO:0048015 phosphoinositide-mediated signaling (5.07e-13) GO:0007049 cell cycle (1.79e-09) GO:0006260 DNA replication (5.37e-08) GO:0004674 protein serine/threonine kinase activity (1.09e-07) GO:0005524 ATP binding (1.29e-07) GO:0005634 nucleus (1.93e-07) GO:0006468 protein amino acid phosphorylation (1.01e-06)
05 low	GO:0045012 MHC class II receptor activity (8.59e-08) GO:0006968 cellular defense response (5.07e-07) GO:0006955 immune response (6.9e-07)
07 high	GO:0005201 extracellular matrix structural constituent (3.27e-20) GO:0005578 extracellular matrix (sensu Metazoa) (2.94e-15) GO:0005581 collagen (1.76e-13) GO:0006817 phosphate transport (2.79e-12) GO:0007517 muscle development (8.27e-10) GO:0005518 collagen binding (1.1e-07) GO:0008201 heparin binding (8.6e-07) GO:0007155 cell adhesion (1.55e-06) GO:0005509 calcium ion binding (2.16e-06)
08 high	GO:0007283 spermatogenesis (3.91e-10)
11 low	GO:0042742 defense response to bacteria (1.09e-06)
13 high	GO:0019884 antigen presentation, exogenous antigen (3.23e-16) GO:0019886 antigen processing, exogenous antigen via MHC class II (5.1e-16) GO:0045012 MHC class II receptor activity (5.1e-16) GO:0006955 immune response (1.28e-15)
13 low	GO:0006955 immune response (9.9e-16) GO:0042110 T cell activation (1.15e-10) GO:0007166 cell surface receptor linked signal transduction (1.64e-10) GO:0004888 transmembrane receptor activity (1.25e-09) GO:0006968 cellular defense response (2.05e-07) GO:0019735 antimicrobial humoral response (sensu Vertebrata) (4.81e-07) GO:0005515 protein binding (1.4e-06)
14 low	GO:0005615 extracellular space (4.94e-24) GO:0005319 lipid transporter activity (5.77e-11) GO:0007596 blood coagulation (6.05e-11) GO:0006953 acute-phase response (1.47e-10) GO:0004867 serine-type endopeptidase inhibitor activity (6.14e-10) GO:0004263 chymotrypsin activity (4.83e-09) GO:0004295 trypsin activity (7.86e-09) GO:0006810 transport (1.71e-08) GO:0016042 lipid catabolism (6.48e-08) GO:0005576 extracellular region (8.91e-08) GO:0008201 heparin binding (1.05e-07) GO:0006508 proteolysis and peptidolysis (4e-07) GO:0006869 lipid transport (1.14e-06)
15 high	GO:0005200 structural constituent of cytoskeleton (3.17e-12) GO:0005882 intermediate filament (1.99e-10) GO:0008544 epidermis development (4.44e-10) GO:0007517 muscle development (9.24e-10)
15 low	GO:0008201 heparin binding (1.71e-07) GO:0005201 extracellular matrix structural constituent (3.95e-07)
16 low	GO:0005179 hormone activity (4e-10) GO:0005576 extracellular region (1.51e-07) GO:0007565 pregnancy (1.57e-07)
17 high	GO:0006955 immune response (6.06e-07)
18 high	GO:0005882 intermediate filament (1.47e-11) GO:0005200 structural constituent of cytoskeleton (5.67e-10) GO:0005615 extracellular space (2.74e-08) GO:0008544 epidermis development (1.75e-07) GO:0005319 lipid transporter activity (9.61e-07) GO:0005198 structural molecule activity (1.72e-06)
19 high	GO:0005200 structural constituent of cytoskeleton (2.05e-07)
20 high	GO:0005179 hormone activity (1.88e-08)
21 low	GO:0007517 muscle development (7.01e-27) GO:0008307 structural constituent of muscle (4.15e-23) GO:0003779 actin binding (6.2e-14) GO:0006941 striated muscle contraction (5.69e-10) GO:0005859 muscle myosin (1.99e-08)
22 high	GO:0016042 lipid catabolism (9.41e-08) GO:0004263 chymotrypsin activity (1.94e-06)
23 high	GO:0019825 oxygen binding (1.9e-08) GO:0005344 oxygen transporter activity (2.67e-07) GO:0015671 oxygen transport (3.8e-07)
24 low	GO:0007517 muscle development (8.02e-15) GO:0008307 structural constituent of muscle (2.11e-12) GO:0003779 actin binding (3.61e-09) GO:0005319 lipid transporter actiGO:0005856 cytoskeleton (1e-06)
26 high	GO:0005576 extracellular region (3.7e-10) GO:0005319 lipid transporter activity (2.31e-08) GO:0006869 lipid transport (9.09e-08)
26 low	GO:0008307 structural constituent of muscle (1.19e-08) GO:0005344 oxygen transporter activity (2.67e-07) GO:0015671 oxygen transport (3.8e-07)
29 high	GO:0005615 extracellular space (1.8e-06)
32 low	GO:0005576 extracellular region (3.34e-15) GO:0005615 extracellular space (6.56e-07) GO:0005179 hormone activity (2.15e-06)
35 low	GO:0007585 respiratory gaseous exchange (4e-09)
39 high	GO:0005576 extracellular region (2.35e-07)
40 high	GO:0005179 hormone activity (8.22e-08) GO:0005576 extracellular region (9.39e-07)
42 high	GO:0005179 hormone activity (8.22e-08) GO:0005576 extracellular region (9.39e-07)

**Table 7:** Diabetes dataset covariates identified as correlated with principal components. Results of a search for covariates with value distributions having significant differences in either *UP*_*n *_vs. *DOWN*_*n*_, {*UP*_*n*_+*FLAT*_*n*_} vs. *DOWN*_*n*_, or *UP*_*n *_vs. {*FLAT*_*n*_+*DOWN*_*n*_} are shown below. An "X" indicates the covariate's values varied significantly in the corresponding principal component's sample partitioning. 11 (out of 53) covariates were identified at a 1% significance level (*minSetSize *= 5) in 10 of the first 20 principal components. To investigate further, corresponding plots of covariate value distributions within sample partitions can be generated, such as the covariate plots for PC_14 _(see Figure 5).

	Principal Component Number																			
	
	1	2	3	4	5	6	7	8	9	10	11	12	13	14	15	16	17	18	19	20
Cap_(mm2)		X																		
Cap__Type2b_(mean_n)											X									
Centroid_(Using_34_OXPHOS-CR_Genes)								X												
Insulin_0														X						
Patient_#				X																
Type1_Min_Area_(um2)									X											
Type2a_(n)							X													
Type2a_Min_Area_(um2)																X				
Type2b_(%)														X						
Type2b_(n)			X																	
UQCRB_(209065_at)						X														
