# Supplementary figures and images for: Mining gene expression data by interpreting principal components
Source: BMC Bioinformatics. 2006 Apr 7;7:194. doi: 10.1186/1471-2105-7-194 (PMC1501050; doi:10.1186/1471-2105-7-194)

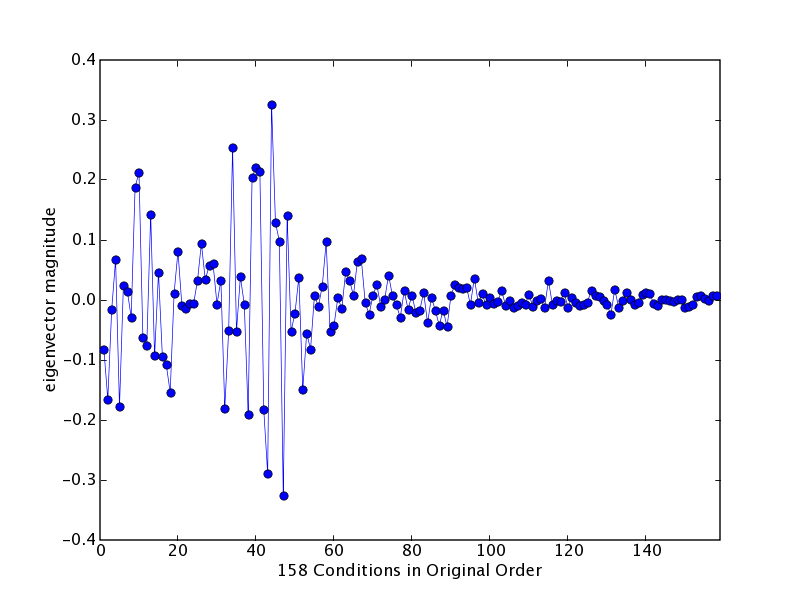

Supplement: Additional File 3 — The supplemental files provided with this publication are only a representative set of those generated by the PCA interpretation software. The complete collections of PCA interpretation results for both the GNF and diabetes datasets are provided as a supplement to this publication at [35]. Trajectory plot of the PC7 eigenvector, or "eigen-condition". [file 1471-2105-7-194-S3.png]

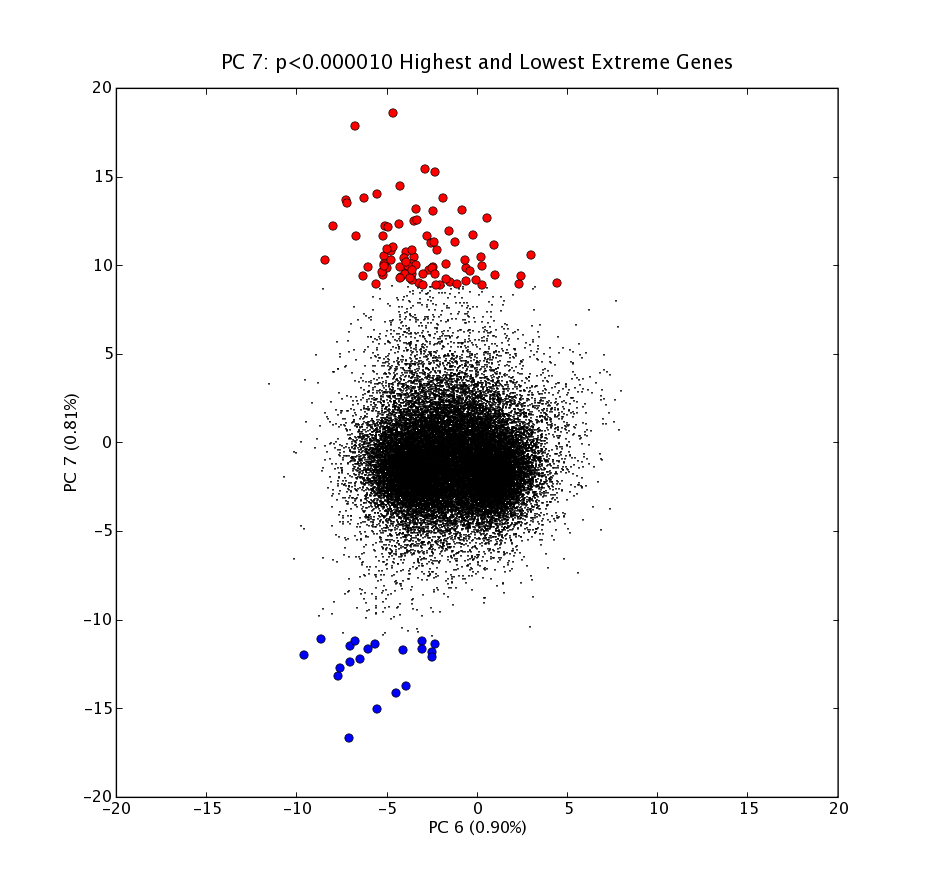

Supplement: Additional File 4 — The supplemental files provided with this publication are only a representative set of those generated by the PCA interpretation software. The complete collections of PCA interpretation results for both the GNF and diabetes datasets are provided as a supplement to this publication at [35]. Scatter plot of gene probe expression levels projected onto PC6 vs. PC7 space. The PC7 high and low extreme gene sets are highlighted in red and blue colors, respectively. [file 1471-2105-7-194-S4.png]

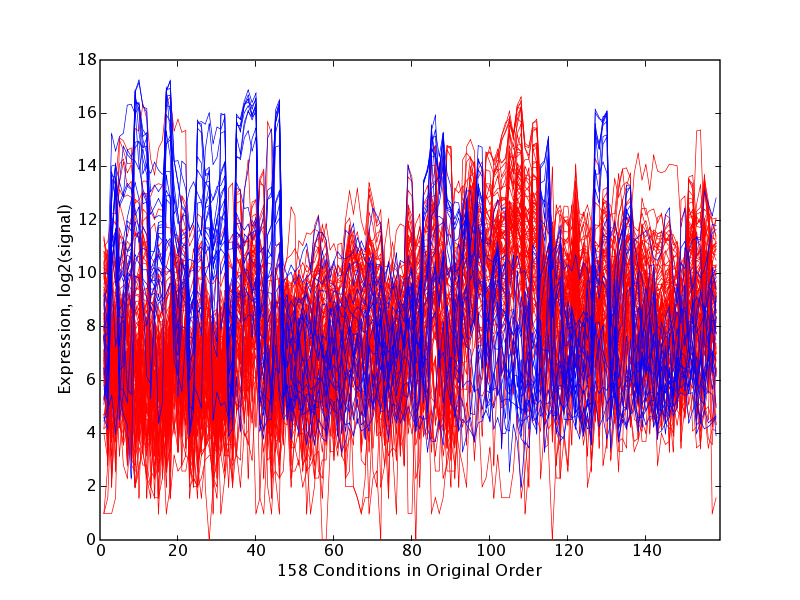

Supplement: Additional File 5 — The supplemental files provided with this publication are only a representative set of those generated by the PCA interpretation software. The complete collections of PCA interpretation results for both the GNF and diabetes datasets are provided as a supplement to this publication at [35]. Gene trajectory plots for PC7 high and low extreme gene sets with tissues in the order in which the original data were provided. [file 1471-2105-7-194-S5.png]

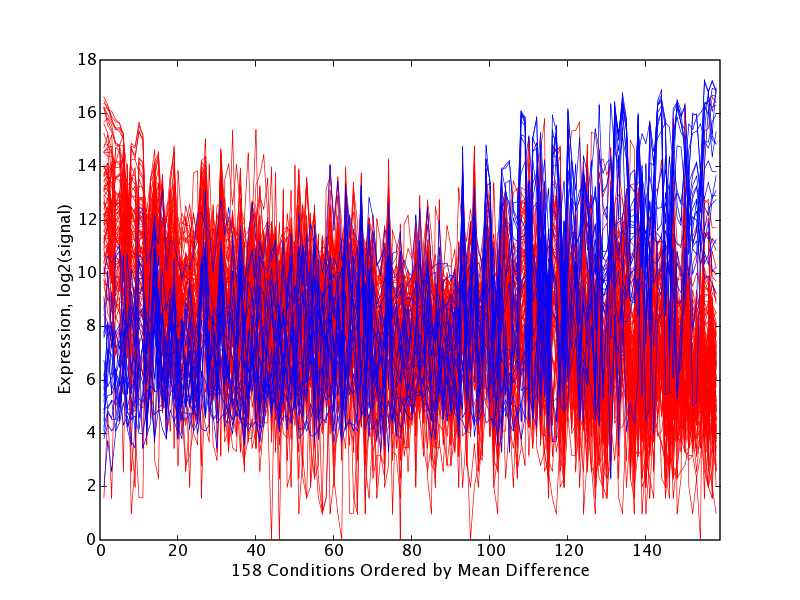

Supplement: Additional File 6 — The supplemental files provided with this publication are only a representative set of those generated by the PCA interpretation software. The complete collections of PCA interpretation results for both the GNF and diabetes datasets are provided as a supplement to this publication at [35]. Gene trajectory plots for PC7 high and low extreme gene sets with tissues ordered by decreasing mean differences, and thus grouped by significance (up group at left, flat group in middle and low group at right). [file 1471-2105-7-194-S6.png]
